# Activation of the Glutamic Acid-Dependent Acid Resistance System in *Escherichia coli* BL21(DE3) Leads to Increase of the Fatty Acid Biotransformation Activity

**DOI:** 10.1371/journal.pone.0163265

**Published:** 2016-09-28

**Authors:** Ji-Min Woo, Ji-Won Kim, Ji-Won Song, Lars M. Blank, Jin-Byung Park

**Affiliations:** 1 Department of Food Science & Engineering, Ewha Womans University, Seoul, 120-750, Republic of Korea; 2 Institute of Applied Microbiology - iAMB, Aachen Biology and Biotechnology - ABBt, RWTH Aachen University, Aachen, Germany; University of Wisconsin Madison, UNITED STATES

## Abstract

The biosynthesis of carboxylic acids including fatty acids from biomass is central in envisaged biorefinery concepts. The productivities are often, however, low due to product toxicity that hamper whole-cell biocatalyst performance. Here, we have investigated factors that influence the tolerance of *Escherichia coli* to medium chain carboxylic acid (i.e., n-heptanoic acid)-induced stress. The metabolic and genomic responses of *E*. *coli* BL21(DE3) and MG1655 grown in the presence of n-heptanoic acid indicated that the GadA/B-based glutamic acid-dependent acid resistance (GDAR) system might be critical for cellular tolerance. The GDAR system, which is responsible for scavenging intracellular protons by catalyzing decarboxylation of glutamic acid, was inactive in *E*. *coli* BL21(DE3). Activation of the GDAR system in this strain by overexpressing the *rcsB* and *dsrA* genes, of which the gene products are involved in the activation of GadE and RpoS, respectively, resulted in acid tolerance not only to HCl but also to n-heptanoic acid. Furthermore, activation of the GDAR system allowed the recombinant *E*. *coli* BL21(DE3) expressing the alcohol dehydrogenase of *Micrococcus luteus* and the Baeyer-Villiger monooxygenase of *Pseudomonas putida* to reach 60% greater product concentration in the biotransformation of ricinoleic acid (i.e., 12-hydroxyoctadec-9-enoic acid (**1**)) into n-heptanoic acid (**5**) and 11-hydroxyundec-9-enoic acid (**4**). This study may contribute to engineering *E*. *coli*-based biocatalysts for the production of carboxylic acids from renewable biomass.

## Introduction

Metabolic engineering allows us to produce a variety of carboxylic acids from renewable biomass compounds such as glucose, xylose, glycerol, and fatty acids. C8 to C18 carboxylic acids can be produced from glucose or glycerol by engineering the fatty acid synthesis pathways of *Escherichia coli* or *Saccharomyces cerevisiae* [1–4]. C5 to C13 carboxylic acids were produced from C18 fatty acids by introducing synthetic enzyme cascades to enable oxidative cleavage of the substrates in *E*. *coli* [5–11] (see S1 Scheme for an example).

However, productivities and product yields of the engineered whole cell-based biocatalysts remained rather low. One of the factors might include the product toxicity to the host cells [12–16]. The toxic effects of the carboxylic acids may lead to damage to the cell membrane and a decrease of the microbial internal pH [12, 14, 17, 18]. The carboxylic acids (e.g., n-octanoic acid) may result in the formation of transient or permanent pores through the interaction with the cellular membranes when entering into the microbial cells in the undissociated form [17]. In particular, these molecules appeared to disrupt the electron transport chain (ETC) and further prevent energy production by uncoupling phosphorylation. The carboxylic acids may also result in intracellular acidification by generating protons within the cells, thereby cause the destabilization of DNA and proteins and the reduction in ATP production due to the decrease of proton gradient.

Another factor to influence the productivities and product yields of engineered whole cell biocatalysts is functional overexpression of the catalytic enzymes. Overexpression of Baeyer-Villiger monooxygenases (BVMOs) in *E*. *coli* or *S*. *cerevisiae*, the key enzymes in synthetic enzyme cascades for the oxidative cleavage of long chain fatty acids (S1 Scheme), remains very difficult [19–25].

*E*. *coli* BL21 is a known and often used host for recombinant protein production [26, 27]. For instance, the BVMOs from *P*. *putida* KT2440 and *P*. *fluorescens* DSM50106 were produced in a functional form [19, 21]. However, *E*. *coli* BL21 is more susceptible to various stresses including acid-induced stress when compared to *E*. *coli* K-12 strains (e.g., MG1655) (see S1 Fig). Therefore, an alternative approach to achieve high productivities and product yields of carboxylic acids would be to improve stress tolerance of *E*. *coli* BL21 strain.

Here, metabolic and genomic responses of *E*. *coli* BL21(DE3) and MG1655 to n-heptanoic acid were intensively examined. The observed differences allowed us to identify factors that influence bacterial tolerance against n-heptanoic acid. Implementing the strategy observed in MG1655 i.e., *dsrA* and *rcsB* over-expression, allowed BL21(DE3) to increase viability in the presence of n-heptanoic acid and subsequently resulting in higher productivity of n-heptanoic acid (**5**) and 11-hydroxyundec-9-enoic acid (**4**) from the C18 fatty acid, ricinoleic acid (**1**) (S1 Scheme).

## Materials and Methods

### *E*. *coli* strains and culture conditions

Strains and plasmids used in this study are listed in S1 Table. *E*. *coli* BL21(DE3) and K-12 MG1655 were cultivated in Lysogeny broth (LB) and Riesenberg medium [28]. The latter was supplemented with 10 g/L glucose. The *E*. *coli* cultures were incubated at 37°C with shaking at 250 rpm (Jeiotech, Daejeon, Korea). When the *E*. *coli* cultures (50 mL in 500 mL baffled flask) reached the exponential growth phases (an optical density of 0.5 at 600 nm, OD_600_), n-heptanoic acid was added to different concentrations (0–10 mM) into the cultivation broth to conduct the growth experiments, *in silico* carbon flux analysis, transcriptome analysis, and measurement of glutamic decarboxylase (GadA/B) activity. *E*. *coli* B, W3110, W, and C strains were grown in the Riesenberg medium containing different concentrations (0–15 mM) of n-heptanoic acid for measurement of specific growth rate and GadA/B activity.

### Quantification of glucose and organic acids

Extracellular concentrations of glucose, acetic acid, lactic acid, and ethanol were measured by high performance liquid chromatography (HPLC) (Waters, Bedford, MA, USA) using an HPX-87H column (Bio-Rad Aminex, Hercules, CA, USA). The column was eluted with 5 mM H_2_SO_4_ at a constant rate of 0.6 mL/min at 50°C. A refractive index detector and a UV dual absorbance detector (Waters, Bedford, MA, USA) were used for detection of the metabolites. UV detection was carried out at 210 nm.

### Constraints-based flux analysis

The constraints-based flux analyses were carried out by using the *E*. *coli* metabolic network model, which consisted of 57 metabolites (including external metabolites) and 58 biochemical reactions. Concentrations of all measured nutrients and products (i.g., glucose, acetic acid, lactic acid, and ethanol) were used to calculate their specific consumption or production rates, which were then specified as the capacity constraints in the model. The cellular objective of the cell growth rate during the exponential growth phase was maximized using linear programming (LP), thereby resulting in a set of metabolic flux distribution corresponding to the optimal phenotype [29]. In the current work, the LP problem was solved using a stand-alone flux analysis program, MetaFluxNet [30]. The specific growth rate measured during the exponential growth phase was compared with the cell growth predicted by the *in silico* model to validate results, as previously reported [31]. The flux data were not normalized to highlight the difference between *E*. *coli* BL21(DE3) and MG1655 strains at the specific glucose uptake rates and carbon fluxes.

The cellular maintenance energy was calculated according to earlier studies [31, 32]. In brief, it was estimated on a basis of NAD(P)H and ATP balances, which were constructed with the production and consumption rates of these cofactors. The production and consumption rates were calculated based on the specific uptake rate of glucose and the intracellular flux distribution.

### Microarray construction

Microarray interrogations were performed using a custom-designed, Agilent-based microarray platform with 4 × 44 K probes per slide (*E*. *coli*_chip; Agilent Design ID: 063245). Total of 4,267 coding sequences of K12 and 4,153 coding sequences of BL21(DE3) were obtained from NCBI Database and 1 probe was designed for each coding sequence using the eArray software according to standard Agilent probe design criteria. We successfully designed 60-mer probes for 8,238 genes using eArray software (http://earray.chem.agilent.com/earray/). Microarrays were manufactured by Agilent Technologies (USA).

### Transcriptome analysis

When *E*. *coli* started to grow exponentially (OD_600_ = 0.5), 3 or 10 mM heptanoic acid was added to the cultures of BL21(DE3) and K-12 MG1655. After 3 h of incubation, the cells were harvested for RNA purification. Total RNA was isolated by using the Qiagen RNeasy kit (Düsseldorf, Germany). For each RNA, the synthesis of target cRNA probes and hybridization were performed using Agilent’s Low Input Quick Amp WT Labeling Kit (Agilent Technology, USA) according to the manufacturer’s instructions. The hybridized microarrays were washed according to the manufacturer’s washing protocol (Agilent Technology, USA). The hybridization images were analyzed using the Agilent DNA microarray Scanner (Agilent Technology, USA) and the data quantification was performed using the Agilent Feature Extraction software 10.7 (Agilent Technology, USA). The average fluorescence intensity for each spot was calculated, while local background was subtracted. All data normalization and selection of fold-changed transcripts were performed using GeneSpringGX 7.3.1 (Agilent Technology, USA). Normalization for the Agilent one-color method was performed, which including ‘Data transformation: Set intensity values which was less than 5.0 to 5.0’ and ‘Per chip Normalization: Each intensity measurement on a microarray was divided by the median intensity of all measurements included in the microarray. The averages of normalized ratios were calculated by dividing the average of control normalized signal intensity by the average of test normalized signal intensity. The categorization of data quality was according the Agilent manual: genes were labeled following the Agilent manual; genes were labeled “present (P)”, “marginal (M)” or “absent (A)” and filtered by selecting only “P” in each experiment. The intensity values lower than the background value were excluded. Genes showing differences in expression levels of ≥2- or ≤0.5-fold were considered to be differentially expressed genes. The microarray data have been deposited in the GEO database (www.ncbi.nlm.nih.bo/geo) and are accessible through GEO Series accession number GSE73640 (http://www.ncbi.nlm.nih.gov/geo/query/acc.cgi?acc=GSE73640).

### Glutamic acid decarboxylase (GadA/B) activity assay

*E*. *coli* cells were harvested 3 h after n-heptanoic acid treatment by centrifugation at 5000 ×g for 15 min at 4°C. The cells were resuspended in 5 mM potassium phosphate buffer (pH 7.3) and sonicated on ice with 10 pulses for 20 sec (10 sec pauses between pulses). Concentration of total protein in each sample was measured by the Bradford assay (Bio-Rad). 50 μL of the cell lysate were then incubated with 100 mM phosphate—citrate buffer (pH 4.6) containing 20 mM L-glutamate, 1 mM pyridoxal 5’-phosphate, and 100 mM sodium chloride at 37°C for 60 min as described previously with some modifications [33]. The reaction was stopped by adding 100 mM pyrophosphate buffer (pH 8.6) then the concentration of gamma-aminobutyric acid (GABA) produced was measured by GABase assay. An aliquot of reaction sample was mixed with GABase solution (100 mM pyrophos-phate buffer (pH 8.6) containing 3.3 mM β-mercaptoethanol, 5 mM α-ketoglutaric acid, 1.25 mM NADP^+^, and 0.13 U/ml of GABase) for 80 min at 25°C. After the GABase reaction, the amount of NADPH produced was determined by measuring absorbance at 340 nm.

### Acid tolerance assays

Tolerance to n-heptanoic acid and low-pH was tested as described [34]. Briefly, overnight cultures were used to inoculate LB medium. The cultures were cultivated to an OD_600_ of 1. Cultures were diluted 1:10 in M9 medium containing the stressor and were exposed for 1 h. Serial dilutions were plated on LB agar before and after exposure to each stress condition and survival frequency was calculated as survival (%) = colony forming units (CFU) (post stress) / CFU(prior stress)×100.

### Cloning of *dsrA* and *rcsB*

Genomic DNA was extracted from *E*. *coli* K12 MG1655 grown in LB medium by using Gspin^™^ Genomic DNA Extraction Kit (Intron biotechnology, Korea). The *dsrA* and *rcsB* genes were PCR-amplified from genomic DNA and subcloned into the *Bam*HI-*Eco*RI (MCS1) and *Nde*I- *Xho*I (MCS2) of the pCOLAduet vector, respectively. Amplified genes were digested using appropriate restriction enzymes and ligated into the vector using T4 DNA ligase (New England Biolabs, Ipswich, MA, USA). Ligated plasmid was introduced into *E*. *coli* DH5α. Transformants were inoculated in 3 mL LB medium and cultivated overnight. Recombinant plasmids were prepared from cultured *E*. *coli* cells using Exprep plasmid purification kit (GeneAll Biotechnology Co, Seoul, Korea).

### Whole cell biotransformation

The whole-cell biotransformation was carried out on a basis of our previous work [5]. Briefly, recombinant *E*. *coli* were cultivated in Riesenberg medium at 37°C. The expression of the target genes was induced at 20°C with 0.1 mM IPTG. After cell growth reached stationary phase, the culture pH was adjusted to 8.0 with NaOH. Afterwards, the biotransformation was initiated by adding 15 mM ricinoleic acid and 0.5 g/L tween80. The reaction was performed in a 250 ml flask (working volume: 20 ml) in a shaking incubator at 35°C and 200 rpm.

### Product analysis by GC/MS

The concentrations of remaining carboxylic acids in the medium such as ricinoleic acid, n-heptanoic acid, and ω-hydroxyundec-9-enoic acid were determined as described previously [5]. The reaction medium was mixed with an equal volume of ethyl acetate containing palmitic acid as an internal standard. The organic phase was harvested after vigorous vortexing and was then subjected to derivatization by adding N-methyl-N-(trimethylsilyl) trifluoroacetamide (TMS). The TMS derivatives were analyzed using a Thermo Ultra Trace GC system connected to an ion trap mass detector (Thermo ITQ1100GC-ion Trap MS, Thermo Scientific, and Indianapolis, IN, USA). The derivatives were separated on a non-polar capillary column (30 m length, 0.25 μm film thickness, HP-5MS, Agilent Technologies, Palo Alto, CA, USA). A linear temperature gradient was programmed as 90°C, 5°C/min to 280°C. The injection port temperature was 230°C. Mass spectra were obtained by electron impact ionization at -70 eV. Scan spectra were obtained within the range of 100–600m/z. Selected ion monitoring (SIM) was used for the detection and fragmentation analysis of the reaction products.

## Results

### Effect of n-heptanoic acid on central carbon metabolism of *E*. *coli*

**Fig 1:**
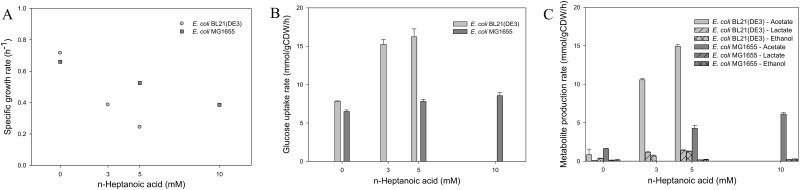
The specific growth rates (A), specific glucose uptake rates (B), and specific metabolite production rates (C) of the *E*. *coli* strains. *E*. *coli* BL21(DE3) (*grey*) and K-12 MG1655 (*dark grey*) were cultivated with different concentrations of n-heptanoic acid at 37°C in a shaking incubator (200 rpm). Values are the mean of more than three independent samples. Bars represent standard error of the mean.

*E*. *coli* BL21(DE3) and K-12 MG1655 were cultivated in a glucose mineral medium with and without n-heptanoic acid to examine the effects of medium chain carboxylic acids on cell growth and carbon metabolism (Fig 1 and S3 Fig). The pH was kept by the buffer in the range of pH 6.3 to 6.8 during all experiments reported. In particular, the culture pH was in the range of pH 6.5±0.05, which is far higher than the pKa value of heptanoic acid (i.e., 4.8) when the samples were prepared for the fluxome and transcriptome analysis. The specific growth rates of the strains were reduced linearly with increasing concentration of the carboxylic acid in the culture broth (Fig 1A). Notably, the specific growth rate of MG1655 was higher, indicating that the strain is more tolerant against the carboxylic acid compared to BL21(DE3). Remarkably, carbon metabolism of MG1655 appeared to remain rather unchanged in the presence of up to 10 mM n-heptanoic acid. However, the specific glucose uptake and acetate production rates of BL21(DE3) were significantly increased (p<0.05) by two- and eighteen-folds, respectively, in the presence of only 5 mM n-heptanoic acid at the identical culture conditions (Fig 1B and 1C).

With an aim to investigate the effects of n-heptanoic acid on carbon metabolism of *E*. *coli* in more detail, *in silico* carbon flux analysis was carried out according to our previous studies [35, 36]. The predicted intracellular carbon flux distribution in the BL21(DE3) and MG1655 growing in the presence of 3 and 10 mM n-heptanoic acid, respectively, indicated higher glucose uptake with the result of increased flux through glycolysis, TCA cycle and acetate formation. The cellular maintenance energy was also increased when exposed to n-heptanoic acid, as previously reported [37]. Overall, carbon metabolism via glycolysis into the TCA cycle as well as into the acetate fermentation pathway was stimulated in the *E*. *coli* strains, especially, in BL21(DE3) in order to afford the increased requirement of the cellular maintenance energy. One of the major reasons may include the enhanced requirement of ATP and maintenance of the proton motive force. The toxicity of weak organic acids because proton transport against the proton gradient is well established [38].

**Fig 2:**
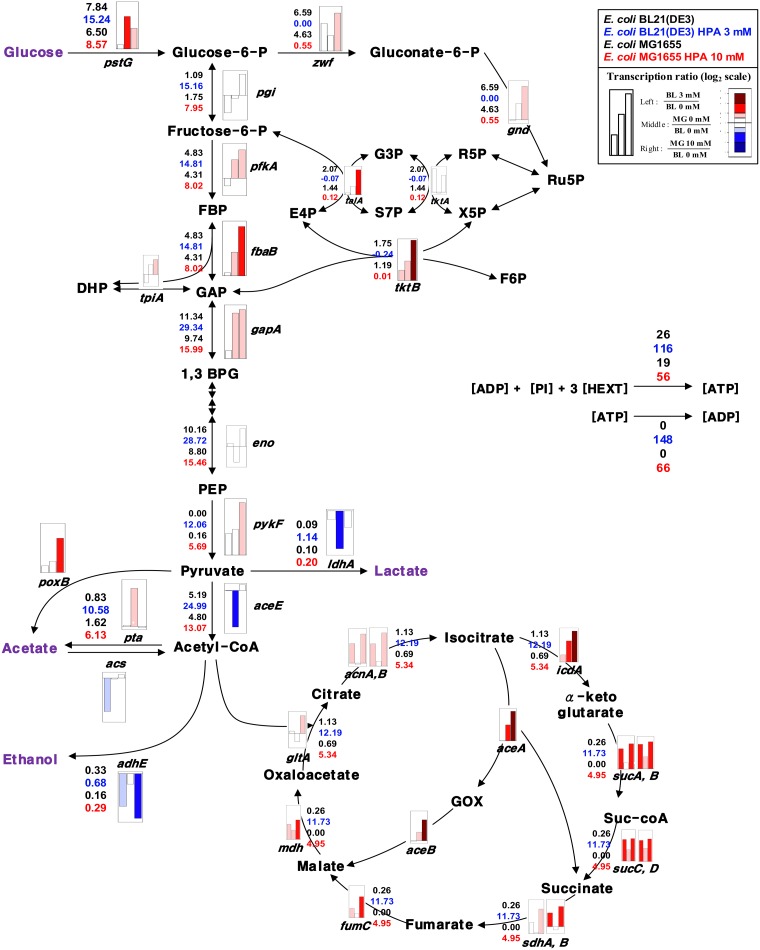
Internal carbon flux distribution in *E*. *coli* BL21(DE3) and K-12 MG1655 strains. The cells were grown in the presence of different concentrations of n-heptanoic acid, as shown in Fig 1. The upper values and third upper values indicate the internal carbon flux distribution in *E*. *coli* BL21(DE3) and K-12 MG1655 strains growing in the absence of n-heptanoic acid. The second upper values and lower values indicate the internal carbon flux distribution in *E*. *coli* BL21(DE3) and K-12 MG1655 strains growing in the presence of n-heptanoic acid. The BL21(DE3) was cultivated with 3 mM n-heptanoic acid, while the MG1655 was with 10 mM n-heptanoic acid. The heptanoic acid concentration is the concentration, in which the specific growth rate of the *E*. *coli* strains reduced to ca. half of the maximum (Fig 1A). Carbon flux distribution was estimated based on stoichiometric constraints using a metabolic network model implemented in MetaFluxNet [30]. The flux data were not normalized to highlight the difference between *E*. *coli* BL21(DE3) and MG1655 strains at the specific glucose uptake rates and carbon fluxes. The normalized data were presented in the Supporting information (S4 Fig). The bars indicate ratios of the expression level of the genes, which products are correspond to the enzymes involved in the reaction step. The first bars indicate the ratio of the gene expression in the BL21(DE3) strain in the absence and presence (3 mM) of n-heptanoic acid. The second bars indicate the ratio of the gene expression between the BL21(DE3) and MG1655 strains in the absence of n-heptanoic acid. The third bars indicate the ratio of the gene expression in the BL21(DE3) strain in the absence of n-heptanoic acid to that in the MG1655 in the presence (10 mM) of n-heptanoic acid.

The transcriptional changes of the central carbon metabolic pathways were rather consistent with the carbon fluxes (Fig 2). In particular, the expression levels of the genes encoding the key enzymes of the TCA cycle (e.g., *icd*, *sucAB*, *gltA*, and *icd*) were markedly increased as was the carbon flux. This result confirmed that the TCA cycle is involved in stress metabolism of *E*. *coli* BL21(DE3) under aerobic conditions.

### Exploring the acid resistance systems of *E*. *coli*

**Fig 3:**
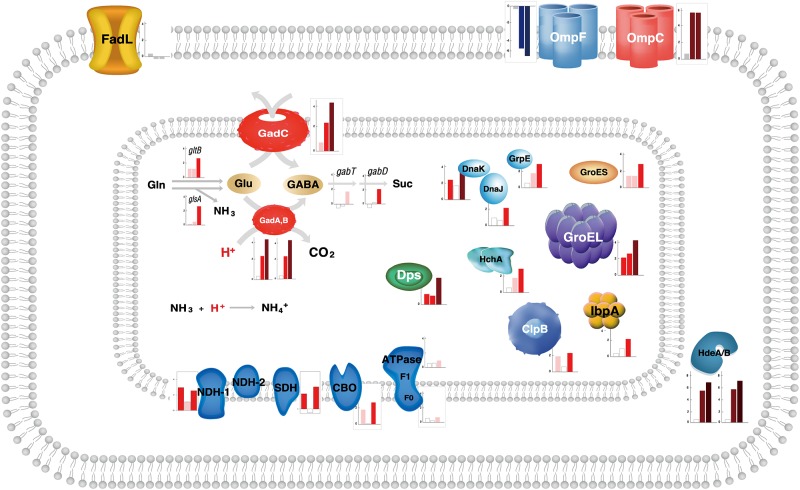
Schematic diagram to show the ratios of expression levels of the genes. The gene products are involved in the acid resistance systems of *E*. *coli* [41] (S3 Table). See Fig 2 for description of the bars.

We have investigated the acid resistance systems to identify factors that influence the tolerance of *E*. *coli* BL21(DE3) to medium chain carboxylic acid (i.e., n-heptanoic acid). First, the oxidative electron transport chain that is implicated in proton efflux during mild acid stress under aerobic growth conditions [39–41] was examined. The transcriptome analysis clearly showed that expression of the core members such as the NADH dehydrogenase I (NDH-I) complex, the succinate dehydrogenase (SDH) complex, and the cytochrome *bo* oxidase (CBO) complex were upregulated at least 2-fold in the presence of n-heptanoic acid in both strains (Table 1, S2 Table and Fig 3). This indicates that glycolysis, the TCA cycle, and the electron transport chain were all activated to pump protons out of the cell, which are transported into the cytoplasm by the carboxylic acid.

**Table 1:** Differential gene expression associated with the acid resistance systems in response to n-heptanoic acid stress.

		Log 2 ratio^1^				
	Gene	Stressed BL21(DE3) / BL21(DE3)^2^	Stressed MG1655 / MG1655^3^	MG1655 / BL21(DE3)	Stressed MG1655 / BL21(DE3)	Stressed MG1655 /Stressed BL21(DE3)
Electron transport chain and ATP synthase	*nuoA*	-0.01	-0.07	**1.02**	0.96	0.97
	*nuoB*	0.09	0.08	0.91	**1.00**	0.90
	*nuoC*	**1.35**	**1.41**	0.92	**2.33**	0.98
	*nuoE*	**1.49**	**1.56**	**1.11**	**2.67**	**1.18**
	*nuoF*	**2.07**	**1.56**	0.50	**2.07**	-0.01
	*nuoG*	**2.95**	**1.45**	**1.09**	**2.54**	-0.41
	*nuoH*	**1.03**	0.43	0.24	0.67	-0.36
	*nuoI*	**1.12**	0.46	0.24	0.69	-0.42
	*nuoJ*	**1.85**	0.76	0.61	**1.37**	-0.48
	*nuoK*	**2.06**	0.72	0.92	**1.63**	-0.42
	*nuoL*	**2.26**	0.61	0.76	**1.37**	-0.88
	*nuoM*	**1.34**	0.45	0.47	0.91	-0.42
	*nuoN*	**1.77**	0.63	0.63	**1.26**	-0.51
	*ndh*	**-1.79**	0.96	**-3.00**	**-2.04**	-0.25
	*sdhC*	-0.25	0.10	-0.53	-0.43	-0.18
	*sdhD*	-0.50	-0.11	-0.12	-0.23	0.27
	*sdhA*	0.99	**2.09**	0.08	**2.17**	**1.18**
	*sdhB*	**2.02**	**3.44**	-0.48	**2.96**	0.93
	*cyoA*	**1.32**	**2.50**	0.07	**2.57**	**1.25**
	*cyoB*	**1.78**	**2.68**	0.07	**2.75**	0.97
	*cyoC*	**1.71**	**2.73**	0.08	**2.82**	**1.11**
	*cyoD*	**2.18**	**2.41**	0.17	**2.57**	0.39
	*cyoE*	**2.41**	**2.06**	0.57	**2.63**	0.22
	*frdA*	0.11	**-2.33**	**2.53**	0.20	0.09
	*frdB*	0.24	**-2.40**	**1.87**	-0.53	-0.76
	*frdC*	0.37	**-1.90**	**3.45**	**1.55**	**1.18**
	*frdD*	0.30	**-1.94**	**1.52**	-0.42	-0.69
	*cydA*	-0.82	**1.40**	**-2.59**	**-1.19**	-0.37
	*cydB*	-0.68	0.58	**-2.09**	**-1.52**	-0.85
	*atpA*	0.53	0.62	0.61	**1.23**	0.69
	*atpD*	0.84	0.62	-0.16	0.46	-0.39
	*atpG*	0.86	0.88	-0.06	0.82	-0.07
	*atpH*	0.60	0.56	0.57	**1.13**	0.55
	*atpC*	0.84	0.55	-0.25	0.30	-0.57
	*atpB*	0.53	0.10	0.14	0.24	-0.29
	*atpF*	0.70	0.77	0.29	**1.06**	0.37
	*atpE*	0.32	0.37	0.21	0.58	0.27
	*atpI*	0.31	0.10	-0.09	0.01	-0.33
FHL complex	*fdhF*	-0.37	**-2.90**	**1.94**	-0.96	-0.59
	*hycD*	-0.74	-0.76	0.71	-0.05	0.69
	*hycC*	-0.68	-0.89	0.58	-0.30	0.37
	*hycF*	-0.83	-0.79	0.08	-0.70	0.12
	*hycG*	-0.45	**-1.22**	**1.95**	0.72	**1.17**
	*hycB*	-0.47	**-1.14**	0.33	-0.80	-0.34
	*hycE*	-0.94	-0.91	-0.10	**-1.01**	-0.07
GDAR and involved systems	*gadA*	0.36	**2.27**	**2.54**	**4.81**	**4.45**
	*gadB*	0.37	**2.28**	**2.56**	**4.84**	**4.46**
	*gadC*	0.79	**2.04**	**2.50**	**4.54**	**3.75**
	*gltB*	**1.16**	**1.28**	**1.17**	**2.44**	**1.28**
	*glsA*	0.11	**2.90**	0.70	**3.60**	**3.49**
	*gabT*	-0.47	**1.77**	-0.14	**1.63**	**2.10**
	*gabD*	-0.78	**1.99**	0.20	**2.18**	**2.96**
GDAR regulation systems	*gadE*	0.31	**1.97**	**1.26**	**3.23**	**2.92**
	*gadW*	0.22	0.70	0.99	**1.69**	**1.47**
	*gadX*	0.16	0.92	0.75	**1.67**	**1.52**
	*rpoS*	0.18	**1.76**	0.56	**2.33**	**2.14**
	*evgS*	0.58	0.06	0.13	0.19	-0.39
	*evgA*	0.15	-0.52	**1.76**	**1.24**	**1.09**
	*ydeO*	0.94	-0.88	**1.77**	0.90	-0.04
Acid fitness island	*gadA*	0.36	**2.27**	**2.54**	**4.81**	**4.45**
	*gadX*	0.14	0.93	0.75	**1.68**	**1.53**
	*gadW*	0.22	0.70	0.99	**1.69**	**1.47**
	*mdtF*	0.40	0.69	0.06	0.76	0.36
	*mdtE*	0.37	**1.12**	-0.04	**1.08**	0.71
	*gadE*	0.31	**1.97**	**1.26**	**3.23**	**2.92**
	*hdeD*	0.27	**1.29**	**1.00**	**2.29**	**2.03**
	*hdeA*	0.26	**1.10**	**5.42**	**6.52**	**6.26**
	*hdeB*	0.30	0.98	**5.78**	**6.76**	**6.46**
	*yhiD*	0.24	-0.51	**1.46**	0.95	0.71
	*yhiF*	0.22	0.16	-0.11	0.06	-0.16
	*slp*	0.44	**1.78**	0.53	**2.30**	**1.86**
Membrane barrier	*ompF*	-0.73	-0.75	**-5.89**	**-6.64**	**-5.92**
	*ompC*	0.48	-0.03	**5.54**	**5.51**	**5.03**
	*fadL*	0.50	0.00	-0.23	-0.24	-0.73
	*cfa*	-0.43	**2.14**	**-1.05**	**1.10**	**1.52**
Universal stress response genes	*dnaK*	**2.52**	**2.37**	**1.98**	**4.35**	**1.83**
	*dnaJ*	0.89	**1.57**	0.61	**2.18**	**1.29**
	*grpE*	0.66	**1.14**	**1.61**	**2.75**	**2.09**
	*groEL*	**2.28**	**2.57**	**2.86**	**5.43**	**3.15**
	*groES*	**1.32**	**1.83**	**1.37**	**3.19**	**1.88**
	*htpG*	0.28	**1.41**	0.38	**1.79**	**1.51**
	*ibpA*	0.35	**1.36**	0.90	**2.26**	**1.91**
	*ibpB*	-0.27	0.72	-0.19	0.53	0.79
	*clpB*	**1.76**	**1.77**	0.45	**2.22**	0.46
	*hchA*	0.66	**1.78**	**1.64**	**3.42**	**2.77**
	*dps*	**1.26**	**2.11**	**1.04**	**3.15**	**1.89**

^1^ Bold font indicates the genes, which expression ratio was upregulated at least 2-fold.

^2^ The expression ratio for genes in *E*. *coli* BL21(DE3), which was incubated in the absence and the presence of n-heptanoic acid. The stressed BL21(DE3) indicates the BL21(DE3) cells, which were incubated in a glucose mineral medium containing 3 mM n-heptanoic acid. The specific growth rate of BL21(DE3) was reduced to ca. 46% of the specific growth rate in the absence of n-heptanoic acid.

^3^ The expression ratio for genes in *E*. *coli* K-12 MG1655, which was incubated in the absence and the presence of n-heptanoic acid. The stressed MG1655 indicates the cells, which were incubated in a glucose mineral medium containing 10 mM n-heptanoic acid. The specific growth rate of MG1655 was reduced to ca. 40% of the specific growth rate in the absence of n-heptanoic acid.

The expression level of the F1 and Fo components of the ATPase, which can also be involved in proton-pumping [41–43], was also increased (Table 1, S2 Table and Fig 3). This could be related to the increased intracellular carbon flux into the acetate fermentation pathway in the cells challenged by the carboxylic acid, because acetate was reported to be produced when cellular ATP availability is low, to enhance ATP synthesis via the acetate kinase [44]. These results indicate that *E*. *coli* suffer from the intracellular acidification during cultivation in a glucose mineral medium containing n-heptanoic acid.

Another mechanism to remove protons inside cells would be to use proton-consuming acid resistance systems [41]. We have examined expression of the two major classes that are the hydrogen-gas-producing formate hydrogen lyase (FHL) complex and the pyridoxal-5-phosphate (PLP)-dependent amino acid decarboxylase acid resistance systems. Expression of the FHL complex remained unchanged with addition of n-heptanoic acid in MG1655 and BL21(DE3) (Table 1 and S2 Table). This may be due to cultivation of the cells under aerobic conditions. On the other hand, expression level of the glutamic acid dependent acid resistance (GDAR) system, which is known as the most effective mechanism that consumes one molecule of H^+^ through decarboxylating glutamate to γ-aminobutyric acid by glutamic acid decarboxylase (GadA/GadB) [45, 46], was markedly increased in the presence of the carboxylic acid in the MG1655 strain (Table 1, S2 Table and Fig 3). Expression of the genes (*gadC*, *glsA* or *gltB*, *gabT* and *gabD*, *hdeA/B*), which products are involved in the GDAR system (Table 1, S2 Table and Fig 3), was also upregulated in MG1655. Highly similar results were reported in a previous study, which examined the effect of octanoic acid on gene expression of *E*. *coli* MG1655 [47], confirming independently our data. In contrast, expression levels of most genes encoding the enzymes involved in the GDAR system, were not influenced in BL21(DE3) (Table 1 and S2 Table). A possible hypothesis is that the lower expression of the GDAR system is one of the factors determining low acid tolerance of BL21(DE3).

Blocking and/or reducing proton and carboxylic acid import could also be an important mechanism in acid tolerance. The first barrier in transport of carboxylic acids may include the porin proteins (e.g., OmpF, OmpC [48]) and the fatty acid transport protein (FadL). There was a little difference in expression level of the FadL between MG1655 and BL21(DE3) (Table 1, S2 Table and Fig 3). However, expression level of the OmpF/C was significantly different between both strains. In particular, the expression level of OmpF, which was reported to facilitate transport of C6 and C8 carboxylic acids into *E*. *coli* [49], was much lower in the MG1655 strain. Thereby, it was assumed that the relatively high expression level of OmpF in the BL21(DE3) could be one of the reasons to the lower tolerance to the carboxylic acid.

Another factor to influence membrane permeability may include the cyclopropane fatty acyl phospholipid synthase *cfa*, which catalyzes the cyclopropanation of unsaturated lipids in bacteria [41]. In *E*. *coli*, cyclopropanation is thought to be involved in long-term survival of non-growing cells and often associated with environmental stresses [50, 51]. Notably, expression level of the *cfa* was increased with n-heptanoic acid only in the MG1655 (Table 1, S2 Table).

Besides, expression behavior of the molecular chaperones was different between the strains (Table 1, S2 Table and Fig 3). Expression of the *dnaKJ-grpE*, *groEL/ES*, and *clpB* were upregulated in both the MG1655 and BL21(DE3), whereas expression of the *ibpA* and *hchA*, which products belong to the Hsp31 system that stabilizes unfolded proteins at low pH, was increased 2.5 and 3.4-fold respectively in the MG1655 only.

### Activation of the GDAR system in BL21(DE3)

There is large difference in expression of genes involved in acid resistance in MG1655 compared to BL21(DE3). Here, we have investigated activation of one of the acid resistance systems (i.e., GDAR) in the BL21(DE3), because the acid resistance of *E*. *coli* strains (e.g., BL21(DE3), B, MG1655, W3110, W, C) appeared to be dependent upon their GadA/B activities (S2 Fig). The first step was to identify the factors to influence expression of the GDAR system in the BL21(DE3) strain. There was no difference in DNA sequence of the genes encoding the key enzymes (i.e., GadA/B, GadC) and the transcription factor of the GDAR (i.e., GadE) compared to the MG1655. However, RcsB, which is an essential component in GDAR regulation by forming a heterodimer with GadE [52, 53], is absent in the BL21(DE3) genome. Another difference was observed in the small RNAs (e.g., DsrA), which are involved in stress metabolism of *E*. *coli*. In particular, DsrA that is known to stimulate the translation of RpoS [54], which is involved in activation of GadE through the GadXW circuit [46, 55], is absent in BL21(DE3). Hence, we reengineered the genetic inventory of MG1655 in BL21(DE3) by introducing *rcsB* and *dsrA* and investigated the new mutant in detail.

**Fig 4:**
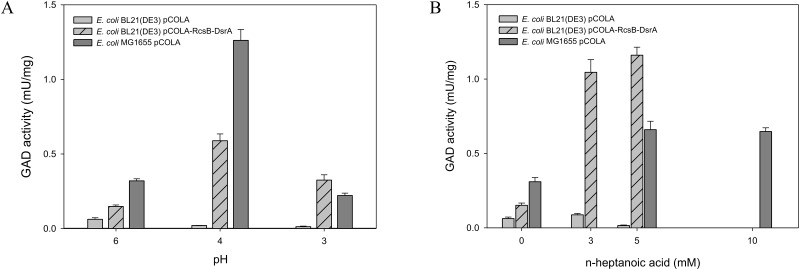
The relationship between GadA/B activity and cultivation pH (A) or n-heptanoic acid concentration (B). The GadA/B activities of the recombinant *E*. *coli* BL21(DE3) pCOLA (empty vector) (*grey bar*), *E*. *coli* BL21(DE3) pCOLA-RcsB-DsrA (*grey bar with diagonal line*) and *E*. *coli* K-12 MG1655 pCOLA (*dark grey bar*) were determined when cells were cultivated at different pH and at different n-heptanoic acid concentration. Values are the mean of more than three independent samples. Bars represent standard error of the mean.

When the *rcsB* and *dsrA* of the MG1655 strain with their native promoters were introduced into *E*. *coli* BL21(DE3), the recombinant cells (i.e., *E*. *coli* BL21(DE3) pCOLA-RcsB-DsrA) showed a GadA/B activity, which is much higher than *E*. *coli* BL21(DE3) pCOLA empty vector at pH 3 or pH 4 (Fig 4A). In fact, the GadA/B activity was comparable to that of *E*. *coli* MG1655 pCOLA. Notably, the GadA/B activity of the recombinant *E*. *coli* BL21(DE3) pCOLA-RcsB-DsrA and *E*. *coli* MG1655 pCOLA was also observed when n-heptanoic acid was added into the culture broth (pH 6.7) (Fig 4B). This result suggested that the GadA/B-based GDAR system was activated when exposed to the carboxylic acid.

**Fig 5:**
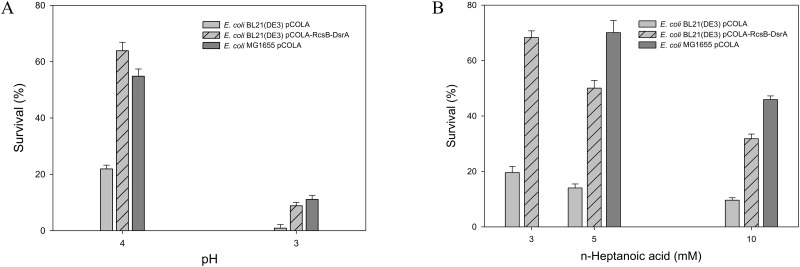
The survival frequency of the recombinant *E*. *coli* strains after 1 h exposure to acidified and n-heptanoic acid added culture medium. The survival frequencies of *E*. *coli* BL21(DE3) pCOLA (empty vector) (*grey bar*), *E*. *coli* BL21(DE3) pCOLA-RcsB-DsrA (*grey bar with diagonal line*) and *E*. *coli* K-12 MG1655 pCOLA (*dark grey bar*) were determined under different cultivation pH (A) and at different n-heptanoic acid concentration (B) as described previously [34]. Values are the mean of more than three independent samples. Bars represent the standard error of the mean.

We have next examined whether the activation of the GDAR system in BL21(DE3) increases acid tolerance. The colony forming unit-based survival frequency was evaluated as previously reported [34]. The survival frequency of the recombinant *E*. *coli* BL21(DE3) pCOLA-RcsB-DsrA was comparable to that of *E*. *coli* MG1655 pCOLA at pH 3 or pH 4, which was 10 (p<0.05) and 3 (p<0.01) times higher than *E*. *coli* BL21(DE3) pCOLA (Fig 5A). This result indicated that the GadA/B-based GDAR system is really involved in acid tolerance of *E*. *coli*. Furthermore, the survival frequency of recombinant *E*. *coli* BL21(DE3) pCOLA-RcsB-DsrA was markedly higher than *E*. *coli* BL21(DE3) pCOLA in the presence of n-heptanoic acid (Fig 5B), indicating that the GDAR system is also one of the key factors to enhance carboxylic acid tolerance. Heptanoic acid tolerance of MG1655 remained however higher, suggesting that other factors are involved in tolerance to n-heptanoic acid stress.

We have also examined the effect of GadA/B activation on carbon metabolism of *E*. *coli* BL21(DE3). The recombinant *E*. *coli* BL21(DE3) pCOLA-RcsB-DsrA and *E*. *coli* BL21(DE3) pCOLA were grown at the conditions identical to the experiments shown in Fig 1. There was no significant difference in the specific glucose uptake rate and metabolite production rates between both strains in the absence of n-heptanoic acid (S5 Fig). However, the specific glucose uptake rate and the acetic acid production rate of the recombinant *E*. *coli* BL21(DE3) pCOLA-RcsB-DsrA was markedly lower than that of *E*. *coli* BL21(DE3) pCOLA at carboxylic acid concentrations of 3 or 5 mM (S6 and S7 Figs). This result indicated that the requirement of cellular maintenance energy was reduced with increased acid tolerance.

### Fatty acid biotransformation activity of *E*. *coli* BL21(DE3) pCOLA-RcsB-DsrA

**Fig 6:**
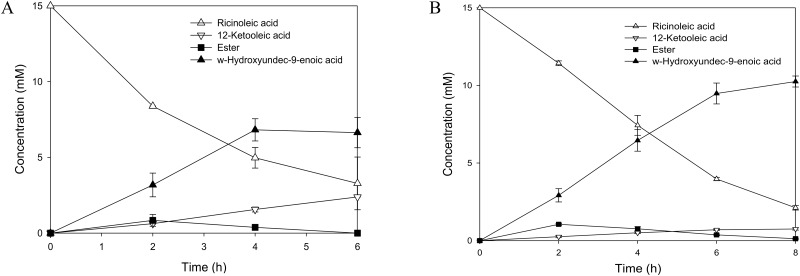
Time course of the biotransformation of ricinoleic acid. Ricinoleic acid (*triangle*) was converted into n-heptanoic acid (**5**) and 11-hydroxyundec-9-enoic acid (**4**) (*solid triangle*) (S1 Scheme) by the recombinant *E*. *coli* BL21(DE3) pACYC-ADH, pET-BVMO, pCOLA (A) and *E*. *coli* BL21(DE3) pACYC-ADH, pET-BVMO, pCOLA-RcsB-DsrA (B). The 12-ketooleic acid (*triangle down*) and ester (*solid square*) are intermediates. The biotransformation was initiated by adding 15 mM of ricinoleic acid, 0.5 g L^−1^ Tween 80, and esterase of *P*. *fluorescens* SIK WI into the culture broth of the strains at the early stationary growth phase (pH 8.0). The cell density was 3.2 g dry cells L^−1^. Values are the mean of more than three independent samples. Bars represent standard error of the mean.

The effect of the GDAR system engineering on the whole-cell fatty acid biotransformation activity was investigated by conducting the bioconversion of ricinoleic acid (i.e., 12-hydroxyoctadec-9-enoic acid (**1**)) into n-heptanoic acid (**5**) and 11-hydroxyundec-9-enoic acid (**4**) (S1 Scheme), which was described in our previous study [15]. When ricinoleic acid was added into the culture broth of the recombinant *E*. *coli* BL21(DE3) pACYC-ADH, pET-BVMO, pCOLA or *E*. *coli* BL21(DE3) pACYC-ADH, pET-BVMO, pCOLA-RcsB-DsrA expressing the alcohol dehydrogenase (ADH) of *Micrococcus luteus* and the Baeyer-Villiger monooxygenase (BVMO) of *Pseudomonas putida*, the final product formation rates of the both cells were similar to at t < 4 h (Fig 6). However, the final product formation rate of *E*. *coli* BL21(DE3) pACYC-ADH, pET-BVMO, pCOLA ceased resulting in an accumulation of the reaction intermediate (**2**) in the culture medium at t > 4 h, when the product concentration and bioconversion yield reached over 6 mM and 45%, respectively (Fig 6A). This might be ascribed to the toxicity of n-heptanoic acid (Fig 5B). In contrast, the final product formation rate of *E*. *coli* BL21(DE3) pACYC-ADH, pET-BVMO, pCOLA-RcsB-DsrA was further maintained resulting in a final product concentration of over 10 mM (conversion yield, 68%), which was 1.6-fold higher as compared to the *E*. *coli* BL21(DE3) pACYC-ADH, pET-BVMO, pCOLA. This value was also over three-folds greater than that of *E*. *coli* MG16655-based biocatalysts expressing the ADH and the BVMO. This result might be ascribed to low expression level of the BVMO in a functional form in the MG16655 strain. Overall, this is consistent with acid tolerance of the cells and functional production of the key enzymes are core factors to influence the fatty acid biotransformation activity.

## Discussion

### Toxic effects of n-heptanoic acid to *E*. *coli*

n-Heptanoic acid is a weak organic acid containing a quite long hydrocarbon chain. Thereby, the carboxylic acid may generate acid-induced stress as much as a kind of solvent stress, as discussed previously [14, 17, 18, 47]. In this study, n-heptanoic acid led to marked changes in carbon metabolisms of *E*. *coli* BL21(DE3); the specific glucose uptake and acetate production rates were substantially increased (p<0.05) by two- and eighteen-folds, respectively, in the presence of 5 mM n-heptanoic acid (Fig 1). Carbon flux into the TCA cycle appeared to be also increased (Fig 2), which was supported by enhanced expression of most genes encoding enzymes of the TCA cycle and electron transport chain (Figs 2 and 3).

The metabolic responses to heptanoic acid of *E*. *coli* MG1655, a strain that is reported for its acid tolerance, were markedly smaller. The specific glucose uptake and acetate production rates remained unchanged (Fig 1). A previous study of the MG1655 strain with octanoic acid challenge showed that the specific glucose uptake rate and carbon flux into the TCA cycle was decreased in the presence of octanoic acid while acetate production rate was increased [56]. In fact, the metabolic responses of MG1655 appeared to be similar to those of *E*. *coli* challenged with organic solvents (e.g., isobutanol, cyclohexanone) [57, 58]. Addition of isobutanol or cyclohexanone into the culture broth resulted in a stimulation of the acetic acid fermentation pathway rather than the TCA cycle via activation of the ArcA/B regulatory system (S3 Table). In addition, in the previous study of MG1655 using octanoic acid it was reported that native acid resistance systems (e.g., the GDAR system) were not involved in supporting growth or alleviating intracellular acidification [47]. It was concluded that circumventing membrane damage is a key for tolerance to the octanoic acid-induced stress. All the results suggest that solvent-like stress of heptanoic acid might be more prominent in MG1655, whereas in BL21(DE3) acid-induced stress appears to be the dominant mechanism.

The specific glucose uptake rate and acetic acid formation rate of *E*. *coli* BL21(DE3) seemed to be related with acid tolerance. Activation of the GDAR system in BL21(DE3) allowed to reduce the specific glucose uptake and acetate production rates by ca. 26% (S6 Fig). Scavenging the intracellular protons via decarboxylation of glutamic acid might attenuate the metabolic burden, which is involved in energy-dependent proton efflux. Furthermore, the final product of stress metabolism (i.e., acetic acid) is also able to generate acid stress to *E*. *coli* [59]. Thereby, increase of acid tolerance may contribute to the reduction of the metabolic burden as well as attenuation of toxic metabolite (i.e., acetic acid) formation.

### Engineering the GDAR system in *E*. *coli* BL21(DE3)

The specific growth rates of the *E*. *coli* strains (i.e., BL21(DE3), B, MG1655, W3110, W, C) were reduced depending on the n-heptanoic acid concentration in the culture medium (S1 Fig). Notably, this reduction in growth was strain dependent. The specific growth rate of *E*. *coli* W was the best maintained with increasing concentration of n-heptanoic acid, whereas that of *E*. *coli* BL21(DE3) decreased most. Another interesting point was the positive correlation between specific growth rate in the presence of acid and the GadA/B activity (S2 Fig). This indicates that the GadA/B activity might play a key role in tolerance of the strains to the n-heptanoic acid stress.

The GDAR system is regulated by the global regulator RpoS, which is involved in the regulation of GadX (S8 Fig) [46]. The GadE, which is under the control of three regulatory circuits (i.e., the signal transduction system EvgA/EvgS, the TrmE circuit, and the GadXW circuit [46]), is also involved in complex regulation of GDAR. RcsB plays a critical role in GDAR regulation by forming a heterodimer with GadE before binding to the regulatory site (GAD box). Another global regulator, the nucleoid associated protein H-NS, enhances degradation of the *rpoS* mRNA [60], and represses the expression of specific regulators of the amino acid—dependent AR systems (GadX, AdiY and CadC [61, 62]), and elements of the GadE regulatory circuits (EvgA, YdeO, GadX, RcsB [61, 63, 64]). The global regulators RpoS and H-NS are in turn regulated by noncoding small RNAs (e.g., DsrA) (S8 Fig) [54, 65]. DsrA is involved in the activation of RpoS translation by binding to the 5’-untranslated region of the *rpoS* mRNA to free up the ribosomal binding site and in regulation of H-NS by increasing *hns* mRNA turnover [65]. Thus, *dsrA* and *rcsB*, which are missing in the BL21(DE3) genome, were introduced to restore a functional the GDAR system in BL21(DE3). Activation of the GDAR system by expressing the *dsrA* and *rcsB* genes with their native promoters in BL21(DE3) allows to enhance tolerance not only to low pH but also to n-heptanoic acid (Fig 4). The acetic acid formation was significantly reduced (p<0.05) when the *dsrA* and *rcsB* genes were expressed additionally (S5 and S6 Figs). These results indicate that the DsrA and RcsB are truly involved in regulation of the GDAR system and thereby increasing acid tolerance of *E*. *coli*.

## Conclusions

*E*. *coli* BL21(DE3) is more susceptible to acid stress than *E*. *coli* K-12 MG1655. Activation of the GDAR system by introducing the *rcsB* and *dsrA* of MG1655 into BL21(DE3) led to increased acid tolerance not only to low pH but also to n-heptanoic acid. Therefore, it was concluded that the GDAR system played a key role in acid tolerance of *E*. *coli*. The higher ricinoleic acid biotransformation activity of the engineered *E*. *coli* BL21(DE3) also indicated that acid tolerance is one of the factors that influence fatty acid biotransformation activity. This study contributes to a deeper understanding of acid tolerance of *E*. *coli* and presents an engineering strategy for acid tolerance applicable for productive whole-cell biotransformation to synthesize carboxylic acids.

## Supporting Information

S1 Fig The specific growth rates of the *E*. *coli* strains. *E*. *coli* BL21(DE3) (blue), B (cyan), MG1655 (red), W3110 (dark green), W (dark yellow), C (pink)) was cultivated in a glucose mineral medium containing different concentrations of n-heptanoic acid.
(TIF)

S2 Fig The relationship between the specific growth rates and GadA/B activities. *E*. *coli* BL21(DE3) (blue), B (cyan), MG1655 (red), W3110 (dark green), W (dark yellow), C (pink)) was cultivated in a glucose mineral medium containing different concentrations of n-heptanoic acid.
(TIF)

S3 Fig Growth profiles of *E*. *coli* BL21(DE3) (A) and MG1655 (B) toward different concentrations of n-heptanoic acid. n-Heptanoic acid was added to zero (solid lines) or 3 mM (dashed lines) into the culture broth of *E*. *coli* BL21(DE3), whereas added to zero (solid lines) or 10 mM (dashed lines) into the culture broth of *E*. *coli* MG1655. The cultures were incubated in a shaking incubator (200 rpm and 37°C).
(TIF)

S4 Fig Internal carbon flux distribution in *E*. *coli* BL21(DE3) and K-12 MG1655 strains. The cells were grown in the presence of different concentrations of n-heptanoic acid, as shown in Fig 1. The upper values and third upper values indicate the internal carbon flux distribution in *E*. *coli* BL21(DE3) and K-12 MG1655 strains growing in the absence of n-heptanoic acid. The second upper values and lower values indicate the internal carbon flux distribution in *E*. *coli* BL21(DE3) and K-12 MG1655 strains growing in the presence of n-heptanoic acid. The BL21(DE3) was cultivated with 3 mM n-heptanoic acid, while the MG1655 was with 10 mM n-heptanoic acid. The flux data were normalized based on the specific glucose uptake rates.
(TIF)

S5 Fig Growth profiles of recombinant *E*. *coli* BL21(DE3) pCOLA (A), *E*. *coli* BL21(DE3) pCOLA-RcsB-DsrA (B), and *E*. *coli* MG1655 pCOLA (C). n-Heptanoic acid was added to zero (solid lines) or 3 mM (dashed lines) into the culture broth of *E*. *coli* BL21(DE3), whereas added to zero (solid lines) or 10 mM (dashed lines) into the culture broth of *E*. *coli* MG1655. The cultures were incubated in a shaking incubator (200 rpm and 37°C).
(TIF)

S6 Fig The specific glucose uptake rates (A) and specific metabolite production rates (B) of *E*. *coli* BL21(DE3) pCOLA (grey bar), *E*. *coli* BL21(DE3) pCOLA-RcsB-DsrA (grey bar with diagonal line), and *E*. *coli* MG1655 pCOLA (dark grey bar). n-Heptanoic acid was added to zero, 3 mM, and 5 mM into the culture broth of *E*. *coli* BL21(DE3), whereas added to zero, 5mM, and 10 mM into the culture broth of *E*. *coli* MG1655 during the exponential growth phase. The culture was incubated in a shaking incubator (200 rpm and 37°C). Values are the mean of more than three independent samples. Bars represent standard error of the mean.
(TIF)

S7 Fig Internal carbon flux distribution in *E*. *coli* BL21(DE3) pCOLA, *E*. *coli* BL21(DE3) pCOLA-RcsB-DsrA, and *E*. *coli* MG1655 pCOLA. The upper values, third upper values, and fifth upper vlaues indicate the internal carbon flux distribution in *E*. *coli* BL21(DE3) pCOLA, *E*. *coli* BL21(DE3) pCOLA-RcsB-DsrA, and *E*. *coli* MG1655 pCOLA growing in the absence of n-heptanoic acid. The second upper values and fourth upper values indicate the internal carbon flux distribution in *E*. *coli* BL21(DE3) pCOLA and *E*. *coli* BL21(DE3) pCOLA-RcsB-DsrA growing in the presence (3 mM) of n-heptanoic acid. The lower values indicate the internal carbon flux distribution in *E*. *coli* MG1655 pCOLA growing in the presence (10 mM) of n-heptanoic acid. Carbon flux distribution was estimated based on stoichiometric constraints using a metabolic network model implemented in MetaFluxNet [41].
(TIF)

S8 Fig The regulation of GDAR system proposed in the previous studies [41, 54]. (TIF)

S1 Scheme Designed biotransformation pathway in our previous study [13]. Ricinoleic acid (**1**) is converted into ω-hydroxyundec-9-enoic acid (**4**) and n-heptanoic acid (**5**).
(TIF)

S1 Table The strains and plasmids used in this study. (DOCX)

S2 Table All genes upregulated at least 2-fold under n-heptanoic acid stress. (DOCX)

S3 Table Differential gene expression associated with acid or solvent resistance systems. (DOCX)
