# Supplementary figures and images for: Activation of the Glutamic Acid-Dependent Acid Resistance System in Escherichia coli BL21(DE3) Leads to Increase of the Fatty Acid Biotransformation Activity
Source: PLoS One. 2016 Sep 28;11(9):e0163265. doi: 10.1371/journal.pone.0163265 (PMC5040553; doi:10.1371/journal.pone.0163265)

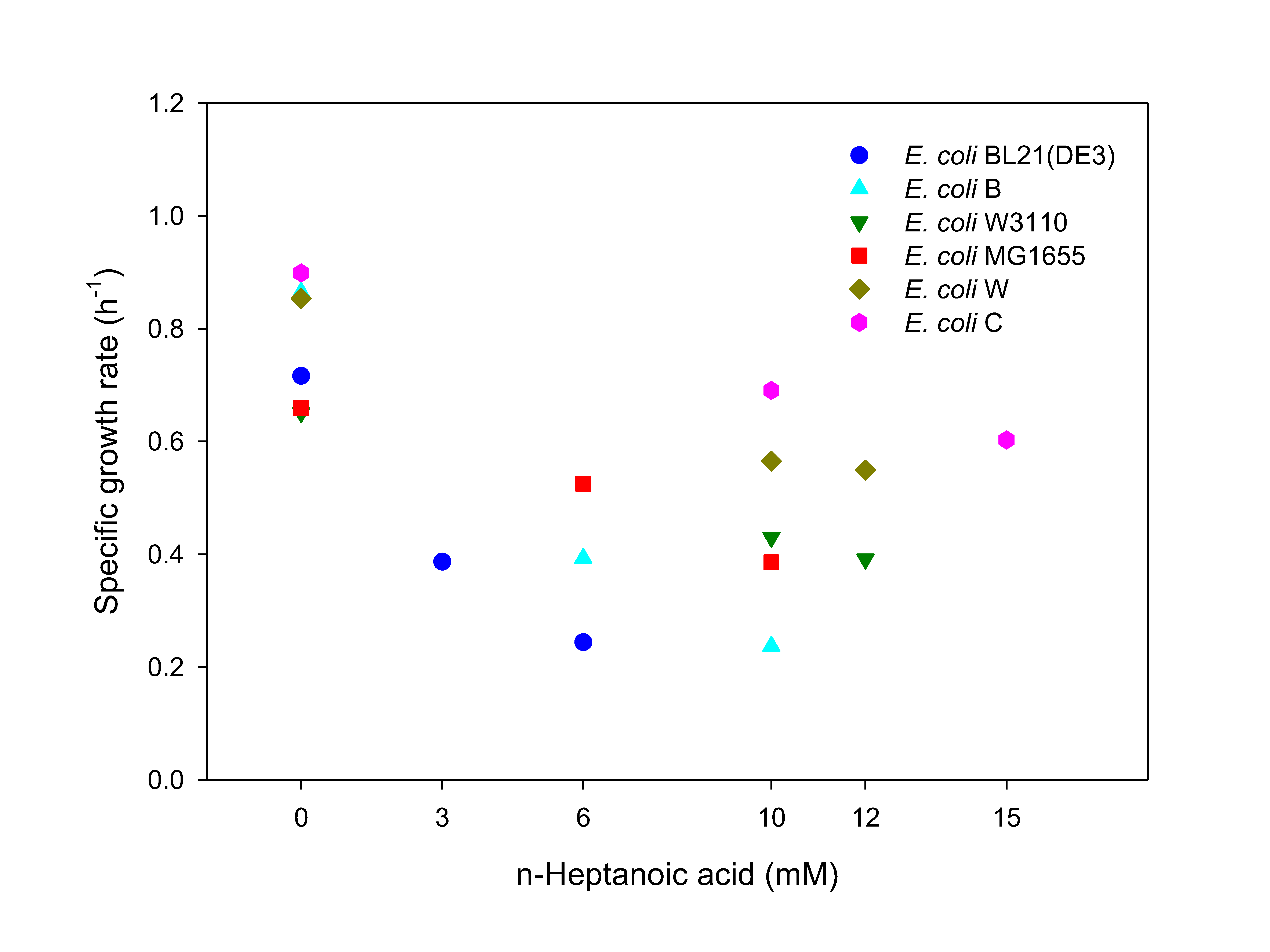

Supplement: S1 Fig — E. coli BL21(DE3) (blue), B (cyan), MG1655 (red), W3110 (dark green), W (dark yellow), C (pink)) was cultivated in a glucose mineral medium containing different concentrations of n-heptanoic acid. (TIF) [file pone.0163265.s001.tif]

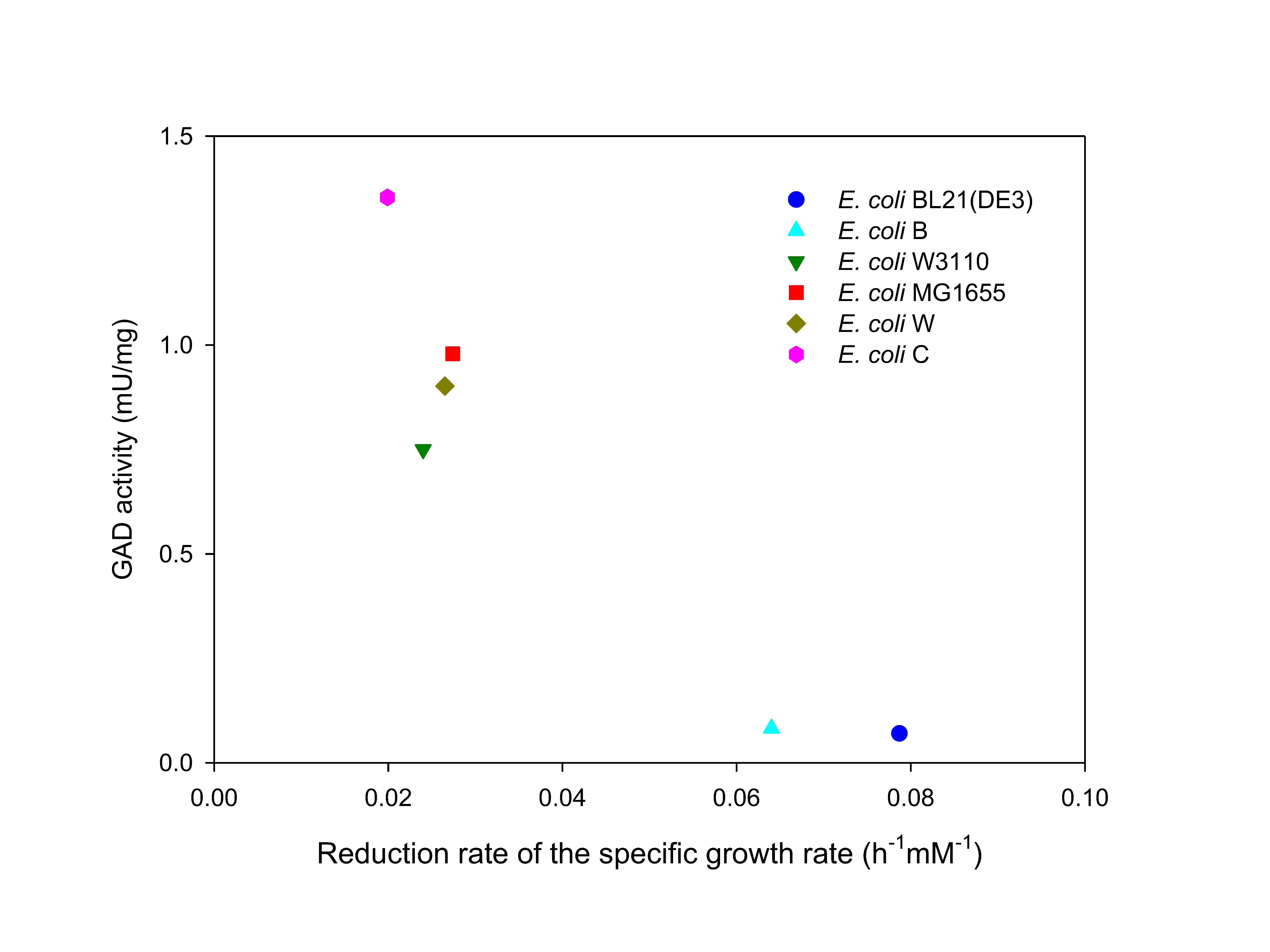

Supplement: S2 Fig — E. coli BL21(DE3) (blue), B (cyan), MG1655 (red), W3110 (dark green), W (dark yellow), C (pink)) was cultivated in a glucose mineral medium containing different concentrations of n-heptanoic acid. (TIF) [file pone.0163265.s002.tif]

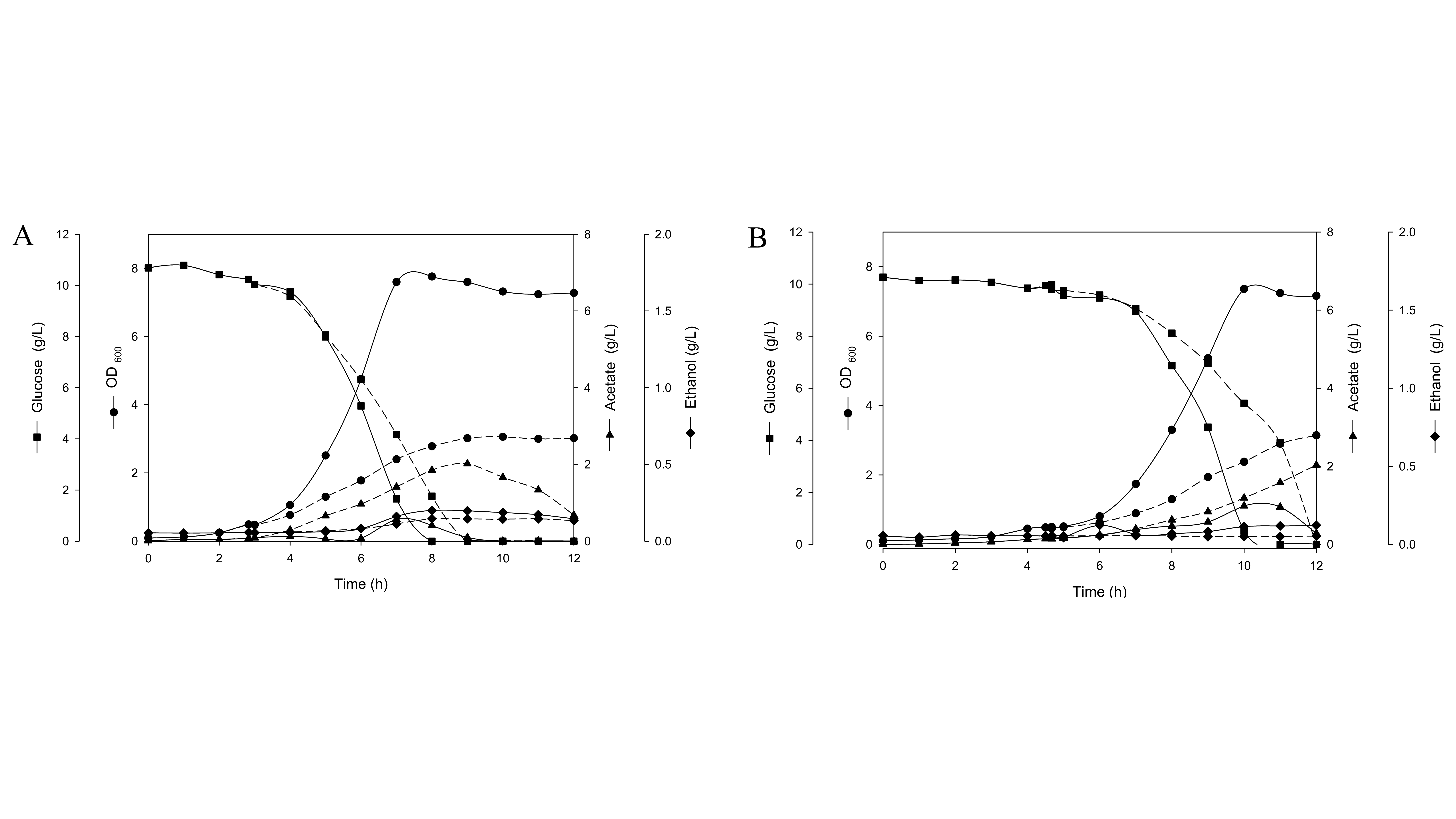

Supplement: S3 Fig — n-Heptanoic acid was added to zero (solid lines) or 3 mM (dashed lines) into the culture broth of E. coli BL21(DE3), whereas added to zero (solid lines) or 10 mM (dashed lines) into the culture broth of E. coli MG1655. The cultures were incubated in a shaking incubator (200 rpm and 37°C). (TIF) [file pone.0163265.s003.tif]

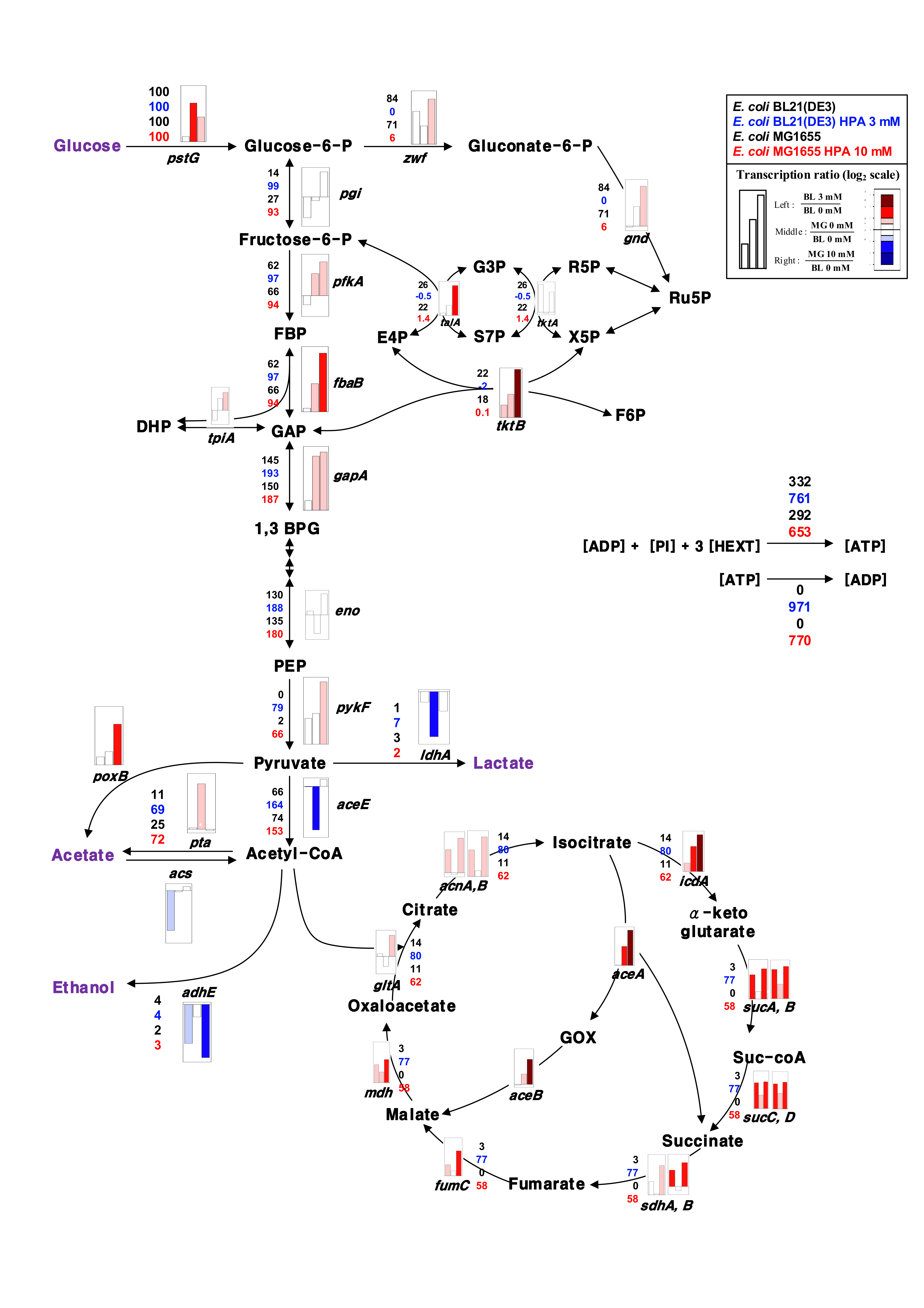

Supplement: S4 Fig — The cells were grown in the presence of different concentrations of n-heptanoic acid, as shown in Fig 1. The upper values and third upper values indicate the internal carbon flux distribution in E. coli BL21(DE3) and K-12 MG1655 strains growing in the absence of n-heptanoic acid. The second upper values and lower values indicate the internal carbon flux distribution in E. coli BL21(DE3) and K-12 MG1655 strains growing in the presence of n-heptanoic acid. The BL21(DE3) was cultivated with 3 mM n-heptanoic acid, while the MG1655 was with 10 mM n-heptanoic acid. The flux data were normalized based on the specific glucose uptake rates. (TIF) [file pone.0163265.s004.tif]

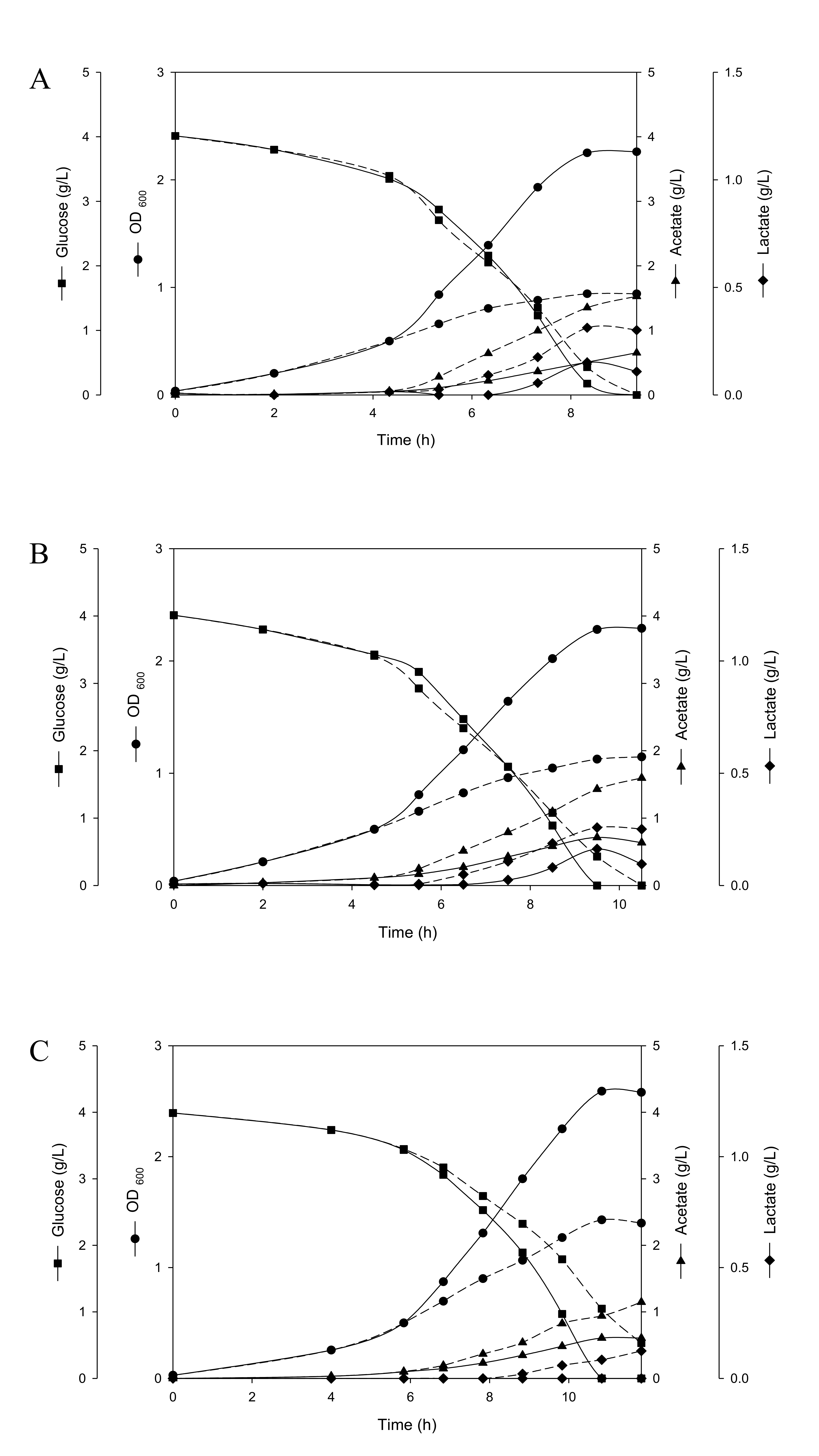

Supplement: S5 Fig — n-Heptanoic acid was added to zero (solid lines) or 3 mM (dashed lines) into the culture broth of E. coli BL21(DE3), whereas added to zero (solid lines) or 10 mM (dashed lines) into the culture broth of E. coli MG1655. The cultures were incubated in a shaking incubator (200 rpm and 37°C). (TIF) [file pone.0163265.s005.tif]

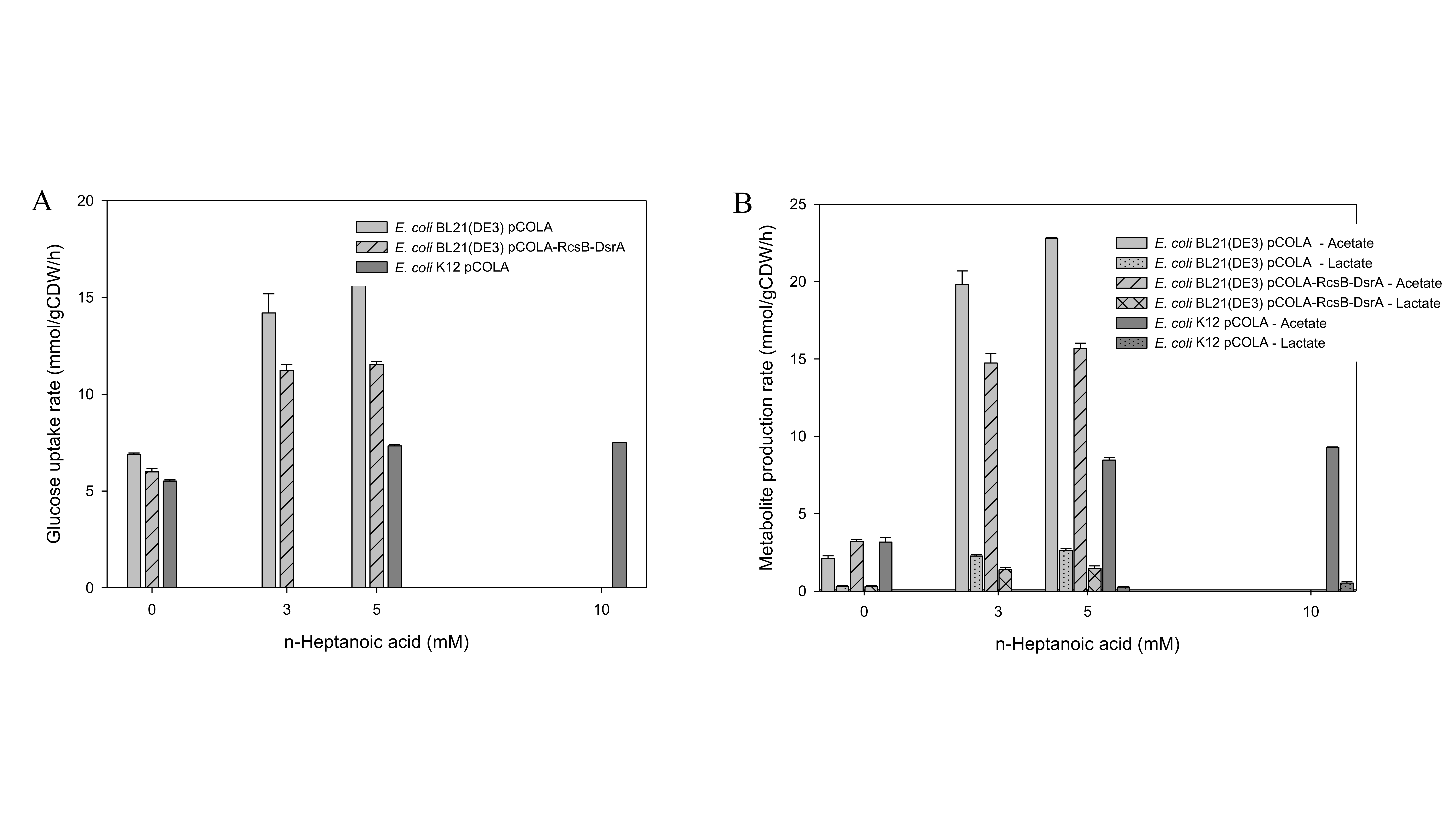

Supplement: S6 Fig — n-Heptanoic acid was added to zero, 3 mM, and 5 mM into the culture broth of E. coli BL21(DE3), whereas added to zero, 5mM, and 10 mM into the culture broth of E. coli MG1655 during the exponential growth phase. The culture was incubated in a shaking incubator (200 rpm and 37°C). Values are the mean of more than three independent samples. Bars represent standard error of the mean. (TIF) [file pone.0163265.s006.tif]

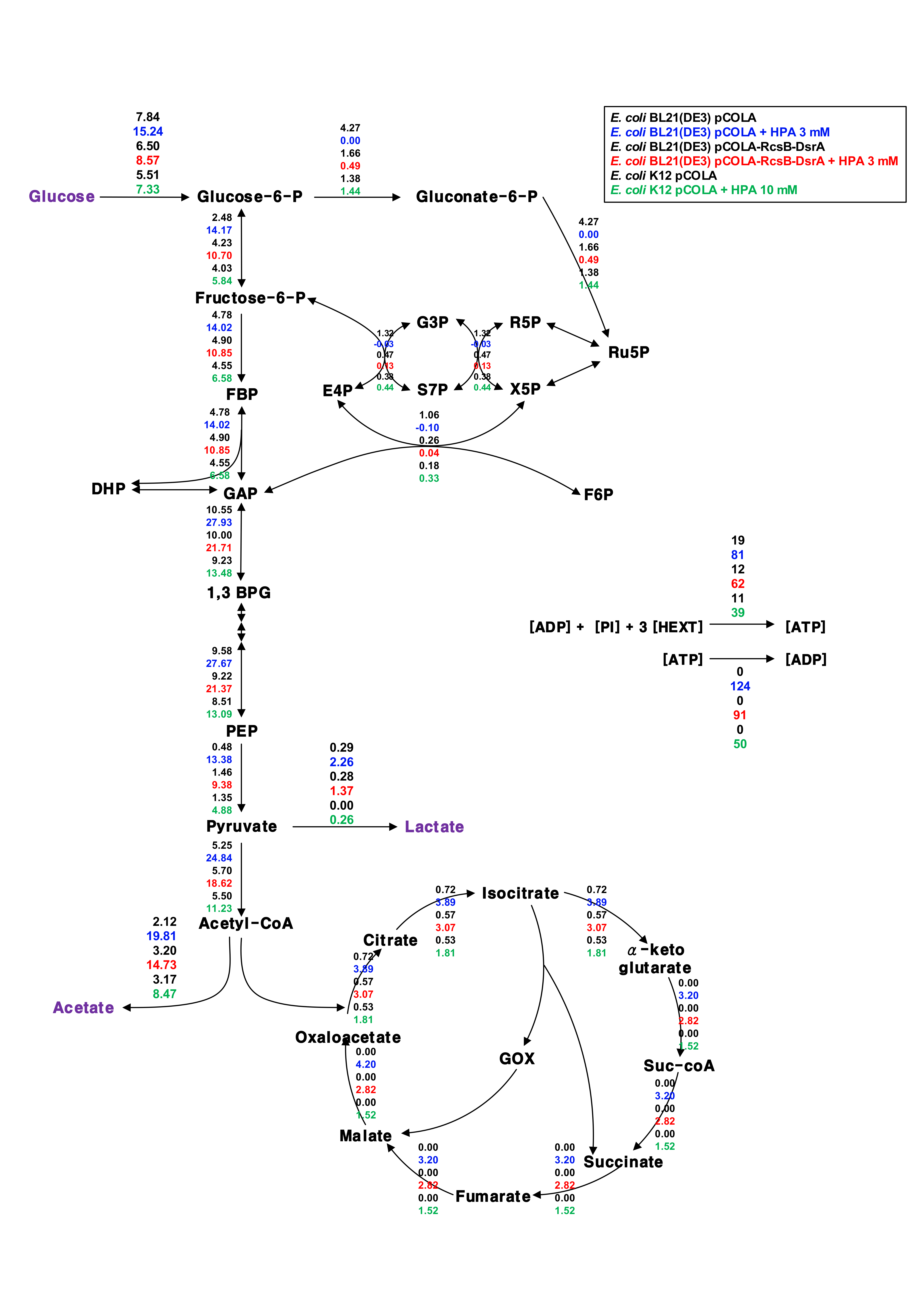

Supplement: S7 Fig — The upper values, third upper values, and fifth upper vlaues indicate the internal carbon flux distribution in E. coli BL21(DE3) pCOLA, E. coli BL21(DE3) pCOLA-RcsB-DsrA, and E. coli MG1655 pCOLA growing in the absence of n-heptanoic acid. The second upper values and fourth upper values indicate the internal carbon flux distribution in E. coli BL21(DE3) pCOLA and E. coli BL21(DE3) pCOLA-RcsB-DsrA growing in the presence (3 mM) of n-heptanoic acid. The lower values indicate the internal carbon flux distribution in E. coli MG1655 pCOLA growing in the presence (10 mM) of n-heptanoic acid. Carbon flux distribution was estimated based on stoichiometric constraints using a metabolic network model implemented in MetaFluxNet [41]. (TIF) [file pone.0163265.s007.tif]

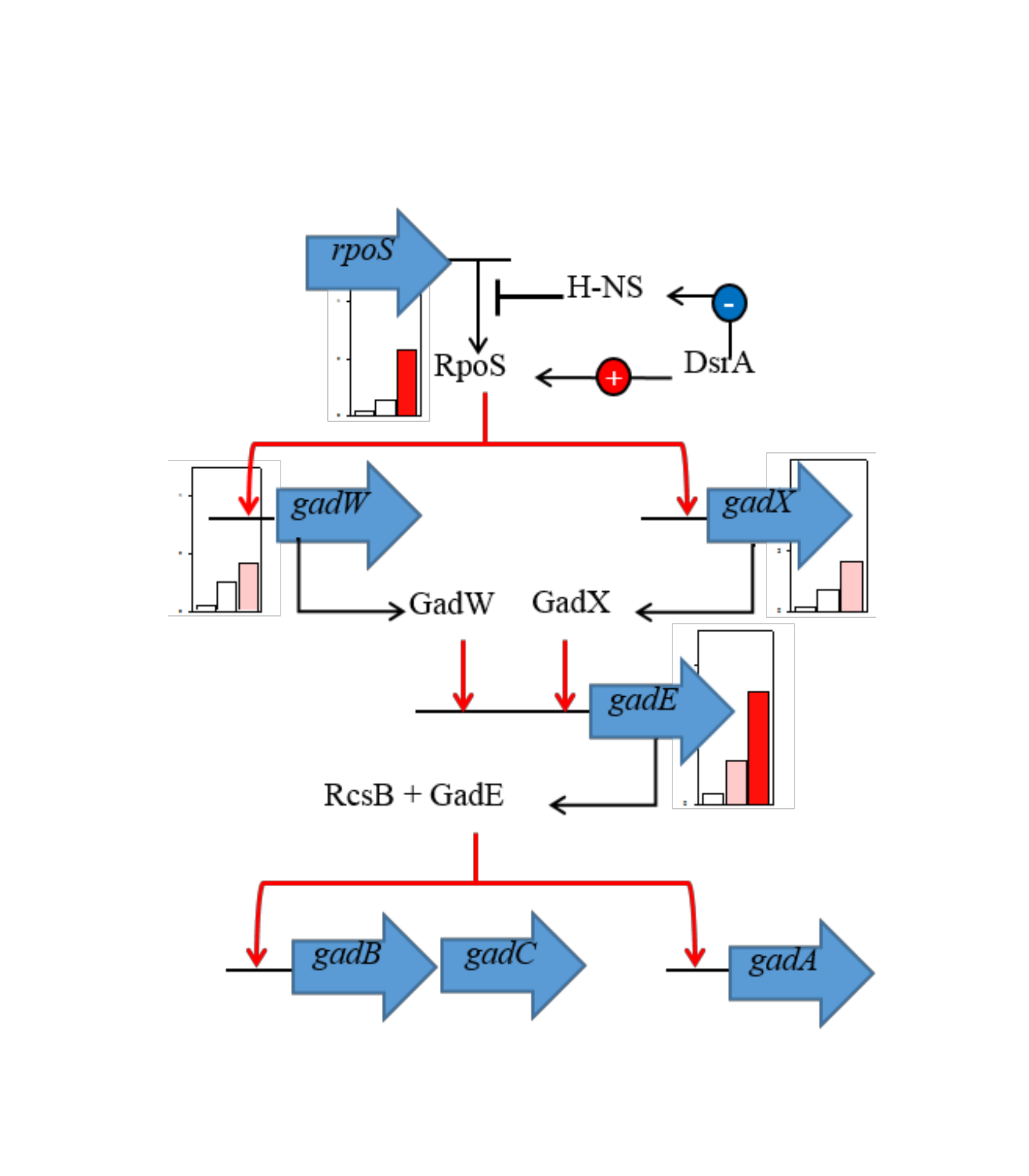

Supplement: S8 Fig — (TIF) [file pone.0163265.s008.tif]

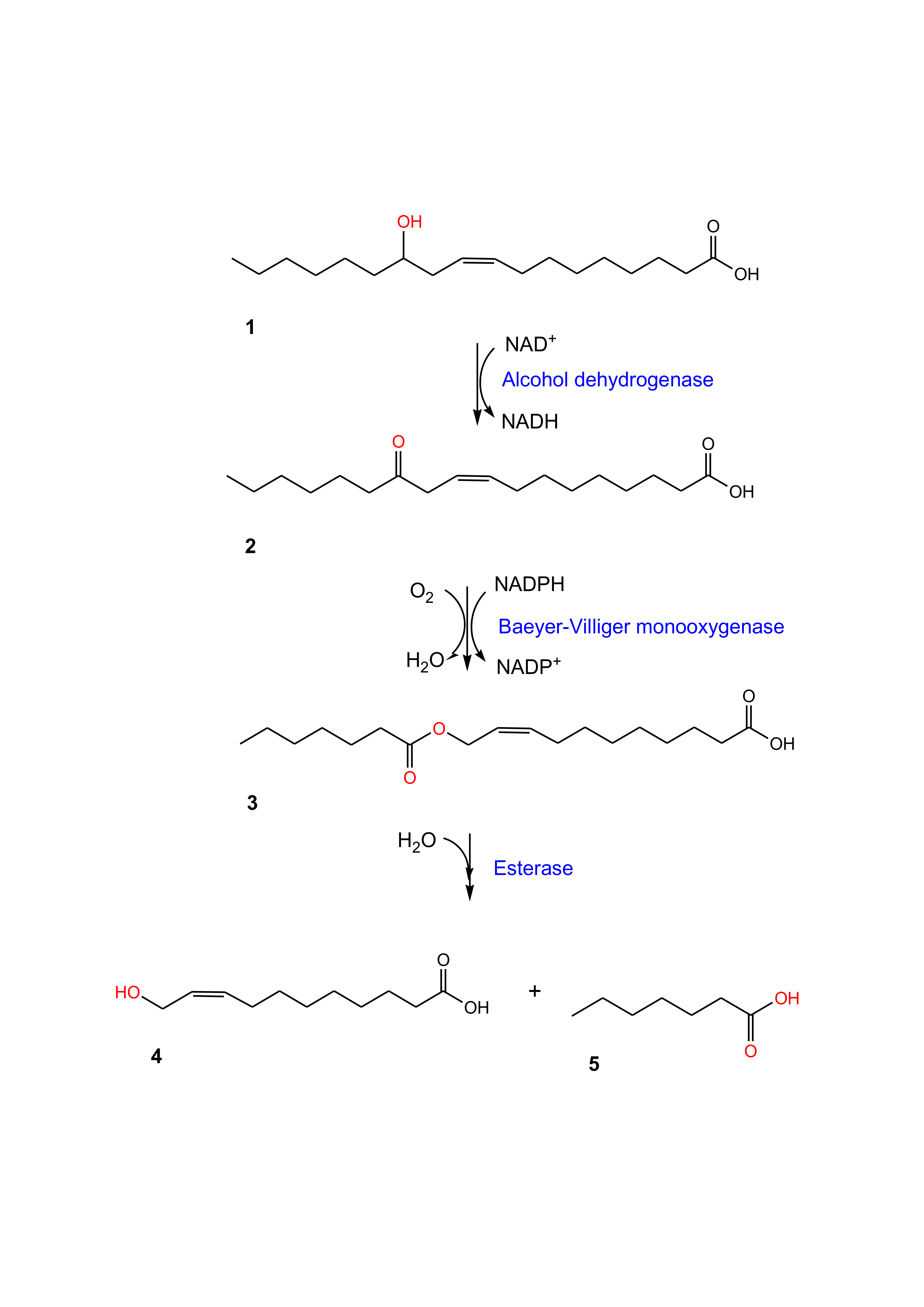

Supplement: S1 Scheme — Ricinoleic acid (1) is converted into ω-hydroxyundec-9-enoic acid (4) and n-heptanoic acid (5). (TIF) [file pone.0163265.s009.tif]
