# Supplementary material for: Activation of the Glutamic Acid-Dependent Acid Resistance System in Escherichia coli BL21(DE3) Leads to Increase of the Fatty Acid Biotransformation Activity
Source: PLoS One. 2016 Sep 28;11(9):e0163265. doi: 10.1371/journal.pone.0163265 (PMC5040553; doi:10.1371/journal.pone.0163265)
Supplement: S1 Table — (DOCX) [file pone.0163265.s010.docx]

**Table S1**. The strains and plasmids used in this study.

| Strain and plasmid | Genotype / containing DNA | Resistance | Source or reference |
| --- | --- | --- | --- |
| *E. coli* MG1655 | F- λ- *ilvG*- rfb-50 rph-1 | NA | ATCC700926 |
| *E. coli* BL21(DE3) | F– *ompT* gal dcm lon hsdSB(rB- mB-) λ(DE3 [*lacI* lacUV5-T7 gene 1 ind1 sam7 nin5]) | NA | Novagen |
| *E. coli* W3110 | F- λ- rph-1 INV(*rrnD*, *rrnE*) | NA | ATCC27325 |
| *E. coli* W | - | NA | KCTC1039 |
| *E. coli* C | - | NA | ATCC8739 |
| *E. coli* B | - | NA | ATCC11303 |
| pCOLADuet™-1 | - | Kan^R^ | Novagen |
| pETDuet™-1 | - | Amp^R^ | Novagen |
| pCOLA-RcsB-DsrA | Carrying the RcsB and DsrA gene of *E. coli* MG1655 | Kan^R^ | This study |
| pACYC-ADH | Carrying the ADH gene of *M. luteus* | Cm^R^ | (Song et al. 2013) |
| pET-BVMO | Carrying the BVMO gene of *P. putida* KT2440 | Amp^R^ | This study |
