# Supplementary material for: Activation of the Glutamic Acid-Dependent Acid Resistance System in Escherichia coli BL21(DE3) Leads to Increase of the Fatty Acid Biotransformation Activity
Source: PLoS One. 2016 Sep 28;11(9):e0163265. doi: 10.1371/journal.pone.0163265 (PMC5040553; doi:10.1371/journal.pone.0163265)
Supplement: S2 Table — (DOCX) [file pone.0163265.s011.docx]

**Table S2. All genes upregulated at least 2-fold under n-heptanoic acid stress.**

| Gene | Log 2 ratio | | | | |
| --- | --- | --- | --- | --- | --- |
|  | Stressed BL21(DE3)  / BL21(DE3)^1^ | Stressed MG1655  / MG1655^2^ | MG1655  / BL21(DE3) | Stressed MG1655  / BL21(DE3) |  |
| *cpxP* | 6.55 | 0.24 | 6.31 | 6.55 |  |
| *copA* | 6.48 | 0.56 | 5.23 | 5.79 |  |
| *zraP* | 5.64 | -2.06 | 1.78 | -0.28 |  |
| *zntA* | 4.37 | 0.92 | -1.55 | -0.63 |  |
| *ycfR* | 4.01 | 6.51 | -0.40 | 6.11 |  |
| *yeeD* | 3.66 | 1.62 | 2.31 | 3.94 |  |
| *yhcN* | 3.47 | 4.84 | -0.67 | 4.17 |  |
| *cysP* | 3.39 | 1.09 | 1.29 | 2.38 |  |
| *cueO* | 3.31 | 0.09 | 2.79 | 2.87 |  |
| *cysD* | 3.07 | 0.64 | 1.83 | 2.47 |  |
| *sucB* | 3.02 | 1.83 | 1.50 | 3.33 |  |
| *yeeE* | 2.97 | 0.61 | 0.52 | 1.13 |  |
| *thrB* | 2.94 | 2.98 | 0.96 | 3.93 |  |
| *nuoG* | 2.95 | 1.45 | 1.09 | 2.54 |  |
| *cysK* | 2.70 | 1.52 | 1.54 | 3.06 |  |
| *sucC* | 2.74 | 1.42 | 1.44 | 2.85 |  |
| *yneI* | 2.71 | 0.84 | -0.14 | 0.69 |  |
| *sucD* | 2.69 | 1.18 | 1.69 | 2.86 |  |
| *cysC* | 2.60 | 0.51 | 2.10 | 2.62 |  |
| *yebE* | 2.57 | -1.76 | 3.49 | 1.73 |  |
| *dnaK* | 2.52 | 2.37 | 1.98 | 4.35 |  |
| *sucA* | 2.46 | 2.27 | 0.29 | 2.56 |  |
| *cyoE* | 2.41 | 2.06 | 0.57 | 2.63 |  |
| *thiS* | 2.39 | 2.54 | 0.49 | 3.03 |  |
| *cysW* | 2.36 | 0.61 | 0.81 | 1.42 |  |
| *thrA* | 2.33 | 2.20 | 0.52 | 2.73 |  |
| *cysN* | 2.31 | 0.74 | 0.48 | 1.21 |  |
| *groEL* | 2.24 | 2.73 | -0.27 | 2.46 |  |
| *nuoL* | 2.26 | 0.61 | 0.76 | 1.37 |  |
| *ybiK* | 2.23 | 0.89 | 1.09 | 1.98 |  |
| *thiF* | 2.22 | 2.30 | 0.32 | 2.62 |  |
| *cyoD* | 2.18 | 2.41 | 0.17 | 2.57 |  |
| *cysU* | 2.14 | 0.31 | 0.88 | 1.19 |  |
| *thiG* | 2.17 | 2.21 | 0.38 | 2.59 |  |
| *cyoC* | 2.12 | 2.92 | -0.07 | 2.86 |  |
| *cysA* | 2.12 | 0.78 | 1.04 | 1.82 |  |
| *ybiJ* | 2.11 | 3.51 | -0.33 | 3.17 |  |
| *rplO* | 2.11 | 1.58 | 1.01 | 2.59 |  |
| *yhcR* | 2.11 | 2.65 | -1.05 | 1.60 |  |
| *rmf* | 2.08 | 1.71 | 1.12 | 2.83 |  |
| *nuoF* | 2.07 | 1.56 | 0.50 | 2.07 |  |
| *nuoK* | 2.06 | 0.72 | 0.92 | 1.63 |  |
| *htpX* | 2.03 | -1.16 | 3.14 | 1.97 |  |
| *sdhB* | 2.02 | 3.44 | -0.48 | 2.96 |  |
| *thiE* | 2.02 | 2.08 | 0.29 | 2.37 |  |
| *marA* | 2.02 | 1.40 | -0.26 | 1.14 |  |
| *yciW* | 2.00 | -0.21 | 0.55 | 0.34 |  |
| *yfiA* | 1.96 | 0.59 | 1.91 | 2.51 |  |
| *yhcQ* | 1.93 | 1.22 | 0.25 | 1.47 |  |
| *marR* | 1.91 | 1.13 | -0.13 | 1.00 |  |
| *mdh* | 1.88 | 1.24 | 0.54 | 1.78 |  |
| *yqjI* | 1.87 | 1.30 | -0.79 | 0.51 |  |
| *nuoJ* | 1.85 | 0.76 | 0.61 | 1.37 |  |
| *rpsB* | 1.81 | 1.13 | 1.73 | 2.86 |  |
| *cyoB* | 1.78 | 2.68 | 0.07 | 2.75 |  |
| *thrC* | 1.78 | 2.73 | 0.16 | 2.89 |  |
| *nuoN* | 1.77 | 0.63 | 0.63 | 1.26 |  |
| *clpB* | 1.76 | 1.77 | 0.45 | 2.22 |  |
| *rplV* | 1.71 | 1.70 | 1.25 | 2.95 |  |
| *thiH* | 1.70 | 1.37 | 0.43 | 1.80 |  |
| *yjfN* | 1.67 | -1.15 | 1.66 | 0.51 |  |
| *marB* | 1.66 | 1.00 | -0.08 | 0.92 |  |
| *cysI* | 1.63 | 0.96 | 1.81 | 2.76 |  |
| *prlA* | 1.63 | 1.01 | 1.32 | 2.32 |  |
| *spy* | 1.60 | -1.74 | 2.61 | 0.87 |  |
| *ygiB* | 1.61 | -0.51 | 1.72 | 1.21 |  |
| *trpB* | 1.59 | 1.14 | 1.43 | 2.57 |  |
| *rpsC* | 1.57 | 1.67 | 0.87 | 2.54 |  |
| *rpoH* | 1.56 | 0.19 | 1.16 | 1.35 |  |
| *rpsH* | 1.55 | 1.35 | 1.50 | 2.85 |  |
| *sodA* | 1.54 | 0.87 | 1.61 | 2.49 |  |
| *osmB* | 1.54 | 1.26 | 2.95 | 4.20 |  |
| *rpsS* | 1.51 | 1.53 | 1.25 | 2.78 |  |
| *mtlR* | 1.49 | 1.99 | -0.20 | 1.79 |  |
| *nuoE* | 1.49 | 1.56 | 0.35 | 1.91 |  |
| *moeA* | 1.47 | 1.21 | 0.71 | 1.92 |  |
| *rpmJ* | 1.46 | 0.85 | 1.27 | 2.12 |  |
| *ompX* | 1.45 | 2.90 | -1.08 | 1.82 |  |
| *rplA* | 1.45 | 1.62 | 1.04 | 2.66 |  |
| *hokD* | 1.45 | 1.13 | -2.03 | -0.89 |  |
| *rpsN* | 1.44 | 1.29 | 1.56 | 2.85 |  |
| *yobB* | 1.42 | -0.82 | 1.79 | 0.96 |  |
| *rplW* | 1.40 | 1.27 | 1.21 | 2.48 |  |
| *rpsM* | 1.39 | 0.77 | 1.35 | 2.12 |  |
| *yhaL* | 1.38 | 0.45 | 1.17 | 1.61 |  |
| *zraR* | 1.37 | 0.18 | -0.02 | 0.15 |  |
| *rplP* | 1.37 | 1.37 | 1.20 | 2.57 |  |
| *rplR* | 1.36 | 1.30 | 1.17 | 2.47 |  |
| *nuoC* | 1.35 | 1.41 | 0.92 | 2.33 |  |
| *rplF* | 1.35 | 1.23 | 1.29 | 2.52 |  |
| *zraS* | 1.34 | -0.47 | -0.28 | -0.75 |  |
| *rplB* | 1.34 | 1.43 | 1.12 | 2.55 |  |
| *nuoM* | 1.34 | 0.45 | 0.47 | 0.91 |  |
| *tauA* | 1.32 | -0.26 | -1.06 | -1.32 |  |
| *cyoA* | 1.32 | 2.50 | 0.07 | 2.57 |  |
| *groES* | 1.32 | 1.83 | 1.37 | 3.19 |  |
| *rplQ* | 1.31 | 1.39 | 0.92 | 2.31 |  |
| *rplK* | 1.31 | 1.59 | 0.71 | 2.30 |  |
| *rplE* | 1.31 | 1.20 | 1.60 | 2.81 |  |
| *nfsA* | 1.31 | 2.44 | -0.63 | 1.81 |  |
| *thrC* | 1.30 | 2.52 | 1.17 | 3.69 |  |
| *yrbD* | 1.29 | 0.93 | 0.91 | 1.84 |  |
| *artI* | 1.29 | 1.03 | 0.75 | 1.78 |  |
| *lpxC* | 1.29 | 1.54 | 0.05 | 1.59 |  |
| *rpmC* | 1.29 | 1.42 | 0.80 | 2.22 |  |
| *rpsO* | 1.29 | 0.52 | 1.35 | 1.87 |  |
| *rpmD* | 1.28 | 1.18 | 0.70 | 1.88 |  |
| *dps* | 1.28 | 2.12 | 1.04 | 3.16 |  |
| *ycbB* | 1.27 | 0.37 | 0.89 | 1.26 |  |
| *thiC* | 1.26 | 1.12 | 0.53 | 1.65 |  |
| *rpmI* | 1.26 | 0.83 | 0.85 | 1.68 |  |
| *rplD* | 1.25 | 1.09 | 1.12 | 2.21 |  |
| *holC* | 1.25 | 1.51 | 0.65 | 2.17 |  |
| *fusA* | 1.25 | 0.79 | 2.29 | 3.08 |  |
| *rpsQ* | 1.24 | 1.35 | 0.86 | 2.20 |  |
| *moaA* | 1.24 | 2.05 | 1.42 | 3.47 |  |
| *yrbE* | 1.23 | 0.43 | 1.21 | 1.64 |  |
| *rplC* | 1.19 | 1.07 | 1.12 | 2.18 |  |
| *fumC* | 1.19 | 2.12 | 0.50 | 2.62 |  |
| *acnB* | 1.19 | 1.50 | 0.25 | 1.74 |  |
| *yccJ* | 1.18 | 1.83 | 0.86 | 2.70 |  |
| *mqo* | 1.17 | 2.49 | -0.32 | 2.18 |  |
| *glgS* | 1.17 | 1.74 | 0.18 | 1.92 |  |
| *rpsV* | 1.17 | 1.14 | 1.59 | 2.73 |  |
| *gltB* | 1.16 | 1.28 | 1.17 | 2.44 |  |
| *ykgE* | 1.16 | -0.15 | -0.12 | -0.27 |  |
| *rplX* | 1.16 | 1.10 | 1.42 | 2.52 |  |
| *sodB* | 1.14 | 0.91 | -0.12 | 0.79 |  |
| *rpoB* | 1.14 | 1.41 | 0.83 | 2.23 |  |
| *acnA* | 1.13 | 1.79 | -0.10 | 1.69 |  |
| *cysJ* | 1.13 | 0.11 | 1.58 | 1.70 |  |
| *fumA* | 1.12 | 1.56 | -0.12 | 1.45 |  |
| *mlaC* | 1.12 | 1.12 | 0.89 | 2.01 |  |
| *nuoI* | 1.12 | 0.46 | 0.24 | 0.69 |  |
| *ydeH* | 1.10 | -0.81 | 2.12 | 1.31 |  |
| *rpsD* | 1.10 | 0.65 | 1.23 | 1.88 |  |
| *ykgG* | 1.09 | 0.10 | -0.04 | 0.06 |  |
| *gltD* | 1.09 | 1.03 | 1.87 | 2.90 |  |
| *rpsK* | 1.08 | 0.58 | 1.16 | 1.74 |  |
| *tsf* | 1.08 | 1.18 | 1.55 | 2.73 |  |
| *metK* | 1.08 | 1.52 | -0.43 | 1.09 |  |
| *ydiJ* | 1.08 | 2.26 | 0.25 | 2.51 |  |
| *ybaJ* | 1.07 | -0.20 | 2.40 | 2.20 |  |
| *bssR* | 1.07 | 0.74 | 3.86 | 4.59 |  |
| *pal* | 1.06 | 1.44 | -0.28 | 1.16 |  |
| *icd* | 1.06 | 1.45 | 3.10 | 4.55 |  |
| *tktB* | 1.05 | 2.11 | 1.97 | 4.07 |  |
| *argB* | 1.05 | -1.19 | 1.44 | 0.25 |  |
| *rplN* | 1.05 | 0.96 | 1.37 | 2.33 |  |
| *nuoH* | 1.03 | 0.43 | 0.24 | 0.67 |  |
| *cusA* | 1.03 | -0.80 | 0.10 | -0.70 |  |
| *miaA* | 1.03 | -0.19 | 2.11 | 1.92 |  |
| *argH* | 1.03 | -0.68 | 0.30 | -0.38 |  |
| *focA* | 1.02 | -0.02 | 2.96 | 2.94 |  |
| *yliA* | 1.02 | 0.84 | 0.38 | 1.22 |  |
| *ydeP* | 1.02 | -0.42 | 0.56 | 0.14 |  |
| *artM* | 1.01 | 0.45 | 0.73 | 1.19 |  |
| *moaC* | 1.01 | 1.59 | 1.77 | 3.36 |  |
| *safA* | 1.01 | -0.29 | 0.45 | 0.16 |  |
| *yacA* | 1.00 | 1.18 | 0.31 | 1.49 |  |

^1^ The expression ratio for genes in *E. coli* BL21(DE3), which was incubated in the absence and the presence of n-heptanoic acid. The stressed BL21(DE3) indicates the BL21(DE3) cells, which were incubated in a glucose mineral medium containing 3 mM n-heptanoic acid. The specific growth rate of BL21(DE3) was reduced to ca. 46% of the specific growth rate in the absence of n-heptanoic acid.

^2^ The expression ratio for genes in *E. coli* K-12 MG1655, which was incubated in the absence and the presence of n-heptanoic acid. The stressed MG1655 indicates the cells, which were incubated in a glucose mineral medium containing 10 mM n-heptanoic acid. The specific growth rate of MG1655 was reduced to ca. 40% of the specific growth rate in the absence of n-heptanoic acid.
