# Supplementary material for: Activation of the Glutamic Acid-Dependent Acid Resistance System in Escherichia coli BL21(DE3) Leads to Increase of the Fatty Acid Biotransformation Activity
Source: PLoS One. 2016 Sep 28;11(9):e0163265. doi: 10.1371/journal.pone.0163265 (PMC5040553; doi:10.1371/journal.pone.0163265)
Supplement: S3 Table — (DOCX) [file pone.0163265.s012.docx]

**Table S3**. Differential gene expression associated with acid or solvent resistance systems.^1^

|  | Acid stress^2^  (Low pH) | Solvent stress^3^  (Isobutanol) | Carboxylic acid stress  (In this study) | |
| --- | --- | --- | --- | --- |
|  |  |  | MG1655 | BL21(DE3) |
| Changes in the cell membrane | ***cfa***  ***ompC***  *ompF* | ***cfa***  *ompF* | ***cfa*** | - |
| Chaperones | ***dnaKJ-grpE***  ***hdeAB***  ***hchA*** | ***dnaKJ-grpE***  ***hdeAB*** | ***dnaKJ-grpE***  ***groEL/ES***  ***hdeAB***  ***hchA*** | ***dnaK***  ***groEL/ES*** |
| Protecting DNA | ***dps*** | ***dps*** | ***dps*** | ***dps*** |
| Metabolic response  & Energy production | ***nuo* genes**  ***sdhCDAB***  ***cyo* genes**  (ETC)  ***gltA***  ***sucABCD***  ***sdhABCD***  ***fumC***  (TCA cycle) | *nuoABCEFGHIKL*  ***ndh***  *sdhCDAB*  *cyoABCDE*  (ETC)  *atpIBEFHAGDC*  (ATPase)  *gltA, acnA, icd,*  *sucABCD,*  *sdhABCD*  *fumC, mdh*  (TCA cycle) | ***nuo* genes**  ***sdhAB***  ***cyoABCDE***  (ETC)  ***gltA, acnAB, icd, sucABCD, sdhAB, fumAC, mdh***  (TCA cycle) | ***nuo* genes**  *ndh*  ***sdhAB***  ***cyoABCDE***  (ETC)  ***acnAB, icd, sucABCD, sdhB, fumAC, mdh***  (TCA cycle) |
| Amino acid dependent decarboxylase systems  & Deaminase systems | ***gadABC, gadE***  ***gltB***  (GDAR)  ***cadA, adiY***  (ADAR, LDAR)  ***ybaS*** | ***gadBC, gadE***  *gltBDF*  (GDAR) | ***gadABC, gadE***  ***gltB***  (GDAR)  ***ybaS*** | - |

^1^ Italics indicates the genes, which expression level was reduced with the stresses, whereas bold italics indicates the genes, which expression level was increased with the stresses.

^2^ Based on the previous studies [[1-4](#_ENREF_1)].

^3^ Based on the previous study [[5](#_ENREF_5)].

**References**

1. Lund P, Tramonti A, De Biase D. Coping with low pH: molecular strategies in neutralophilic bacteria. FEMS Microbiol Rev. 2014;38(6):1091-125. doi: 10.1111/1574-6976.12076. PMID: 24898062.

2. Maurer LM, Yohannes E, Bondurant SS, Radmacher M, Slonczewski JL. pH regulates genes for flagellar motility, catabolism, and oxidative stress in Escherichia coli K-12. J Bacteriol. 2005;187(1):304-19. doi: 10.1128/JB.187.1.304-319.2005. PMID: 15601715.

3. Kannan G, Wilks JC, Fitzgerald DM, Jones BD, Bondurant SS, Slonczewski JL. Rapid acid treatment of *Escherichia coli*: transcriptomic response and recovery. BMC microbiol. 2008;8:37. doi: 10.1186/1471-2180-8-37. PMID: 18302792.

4. Marzan LW, Hasan CM, Shimizu K. Effect of acidic condition on the metabolic regulation of *Escherichia coli* and its phoB mutant. Arch Microbiol. 2013;195(3):161-71. doi: 10.1007/s00203-012-0861-7. PMID: 23274360.

5. Brynildsen MP, Liao JC. An integrated network approach identifies the isobutanol response network of *Escherichia coli.* Mol Syst Biol. 2009;5:277. doi: 10.1038/msb.2009.34. PMID: 19536200.
